# Physiological evidence of stress reduction during a summer Antarctic expedition with a significant influence of previous experience and vigor

**DOI:** 10.1038/s41598-024-54203-9

**Published:** 2024-02-17

**Authors:** Lucie Ráčková, Tomáš Pompa, Filip Zlámal, Miloš Barták, Daniel Nývlt, Julie Bienertová-Vašků

**Affiliations:** 1 RECETOX, Faculty of Sciences, Masaryk University, Brno, Czech Republic; 2 Department of Physical Activities and Health Sciences - Faculty of Sports Studies, Masaryk University, Brno, Czech Republic; 3 Department of Experimental Biology, Masaryk University, Brno, Czech Republic; 4 Polar-Geo-Lab, Department of Geography, Masaryk University, Brno, Czech Republic

**Keywords:** Predictive markers, Physiology, Psychology, Human behaviour, Environmental impact, Psychology and behaviour

## Abstract

Antarctica provides a unique environment for studying human adaptability, characterized by controlled conditions, limited sensory stimulation, and significant challenges in logistics and communication. This longitudinal study investigates the relationship between stress indicators, with a specific focus on mean sleep heart rate, during a COVID-19 quarantine and subsequent 83 days long summer Antarctic expedition at the J. G. Mendel Czech Antarctic Station. Our novel approach includes daily recordings of sleep heart rate and weekly assessments of emotions, stress, and sleep quality. Associations between variables were analyzed using the generalized least squares method, providing unique insights into nuances of adaptation. The results support previous findings by providing empirical evidence on the stress reducing effect of Antarctic summer expedition and highlight the importance of previous experience and positive emotions, with the novel contribution of utilizing physiological data in addition to psychological measures. High-frequency sampling and combination of psychological and physiological data addresses a crucial gap in the research of stress. This study contributes valuable knowledge to the field of psychophysiology and has implications for expedition planners, research organizations, teams in action settings, pandemic prevention protocols, global crises, and long-duration spaceflight missions. Comprehensive insights promote the well-being and success of individuals in extreme conditions.

## Introduction

Antarctica is a natural laboratory for studies of human adaptability^1–3^. Compared to the heroic era of Antarctic exploration, current expeditions operating mainly in research stations infrastructure are safer, with improved food resources, and offer protection from environmental influences and extremes, such as low temperatures, wind, and altered lighting conditions. Nevertheless, they still possess characteristics of an isolated, confined, and extreme (ICE) environment. Current stressors associated with Antarctic summer expeditions include prolonged travels, confinement in stations or small tents during outdoor camps^4,5^, and altered external light conditions^4,6,7^. The sensory stimulation is low and recreation options minimal. The probability of escape or quick rescue is rather low, yet danger associated with fieldwork is still considerably high. Although the medical doctor is typically a member of a crew, availability of medical care, resupply, and communication with the outer world is very limited. The data bandwidth and power supplies are limited aswell^8^. These conditions make Antarctic summer expeditions suitable analogues for future deep space exploration. However, one of their major advantages as a research platform is a high degree of control over variables that might affect psychosocial processes while also avoiding artificial conditions of the traditional laboratory. Therefore, research in Antarctic expeditions is an ideal platform for answering fundamental questions about human psychophysiology and team dynamics^9–11^.

One of the key questions is, what are the temporal and individual determinants of successful adaptation to a challenging environment, described above. Such knowledge may improve general understanding of human adaptation potential as well as improve the selection processes of individuals best suited to manage stress in ICE conditions. Furthermore, it may appoint new countermeasures minimizing or preventing potential risks and supporting well-being^1,4,9,12,13^. Generally, adaptation to the environment is mediated through the stress process during which various mechanisms on the physiological (e.g., heart rate^14–16^), affective (e.g., anxiety^17^, depression, anger^17^, fatigue^18^), cognitive (e.g., stress appraisal^17,19^) and behavioral (e.g., sleep^20–22^) level interplay. Indicators of these processes have been previously addressed in Antarctic expeditions, although predominantly in overwintering crews^4,23,24^. The handful of summer Antarctic expedition research studies frequently finds evidence of lower sleep quality^25^, probably resulting from the disruption of circadian rhythms^4,26^, and social influences emphasized by the isolated environment^26–28^. Some studies measured heart rate and its variability, finding mostly statistically nonsignificant changes between timepoints^5,29–31^. One study found increase in mean heart rate, increase in high-frequency and decrease in low-frequency heart rate variability^32^. Furthermore, studies report salutogenic effect^4,9^, general emotional stability of individuals^9,33^, and prevalence of positive over negative emotions^9,24^, even in comparison with control groups^26^. Some studies also found positive individual changes^4^, as well as decreases in depression, anxiety, fatigue, and confusion^34^.

The current state-of-the-art still has several gaps. Most of previous studies are only cross-sectional^35^ or time-lagged with great intervals^5,29,30,32,36^, which doesn’t elucidate temporal dynamics in long-duration missions. Use of frequently sampled longitudinal data would add power boost into the statistical analyses of otherwise problematic small samples^13^ while also improve understanding of the variance in dynamic variables, within-person relationships, inter-individual differences or significant predictors of successful adaptation^13,17,37^. Only a few studies have addressed multiple different stress markers and their interaction^9,12,17,37,38^, which is fundamental to understand the interrelationships and adaptive variation in individuals. Such findings would improve our understanding of the positive emotions' role on our physical health and well-being in general^35,39,40^, as well as support early detection of unfavorable behavioral conditions^30^, development of precision medicine and precision health systems^8^, or implementation of countermeasures supporting positive adaptation^9^. At the same time, unobtrusive and non-invasive monitoring of physiology is a useful and objective add-on to self-reported measures^9,10,41^.

From the physiological point of view, heart rate and its variability are the standardly used markers of stress^15^. They depend on the pacemaker activity of sinoatrial node, which is influenced by autonomous nervous system^14–16^ a key component of stress reaction. However, heart rate variability analysis requires continuous signal recordings which can be obtained only using holters or chest belts. These devices may be uncomfortable for participants, reducing their compliance and possibility of frequent monitoring. Nowadays it is possible to use heart rate trackers on wrist worn devices which are more user friendly and less obtrusive, enabling to get daily data from participants^30^. This approach also decreases the effect of “white coat” phenomenon which could be apparent in discrete laboratory assessment^14^. Most of these wrist wearables, however, do not allow to collect heart rate variability data in the absence of cloud synchronization, leaving researchers only to point estimates of heart rate. Nonetheless, even a plain heart rate can be valuable for addressing psychophysiological processes if the data collection adheres to rigorous guidelines which have been previously proposed^14^. Nighttime heart rate has a great advantage because it is spared of extraneous factors that may influence recordings. Due to reduced metabolic needs during sleep, mean heart rate decreases from wakefulness to light sleep and further to deep sleep due to the predominance of *nervus vagus*. During REM phase sympathetic activity increases the heart rate^42,43^. Mean sleep heart rate thus reflects the overall activity of nervous system, circulating hormones, and reflex regulation of cardiorespiratory and baroreceptor inputs^16^.

The aim of this study was to explore the relationships between indicators of stress reaction defined as increase in mean sleep heart rate, increase in negative and decrease in positive emotions, increase of perceived stress, and incidence of subjective sleep quality or sleep length problems in participants of a summer Antarctic expedition in a small and isolated J. G. Mendel Czech Antarctic Station on James Ross Island near the northern tip of Antarctic Peninsula. We were interested in changes of the mean sleep heart rate over the expedition, and their relationship with emotions, perceived stress, indicators of sleep quality and other covariates. Our initial hypothesis was that individuals’ markers of stress response will be decreasing during the summer expedition in accordance to previously observed beneficial effects^4,9^. We used daily measurements of heart rate during sleep using sport trackers in accordance with previously proposed guidelines by Nelson et al.^14^, and weekly assessments using state-of-the-art questionnaires. Data were analyzed using generalized least squares (GLS), adjusted for inter-residual correlation and heteroskedasticity.

## Results

### Mean sleep heart rate trajectory

**Figure 1:**
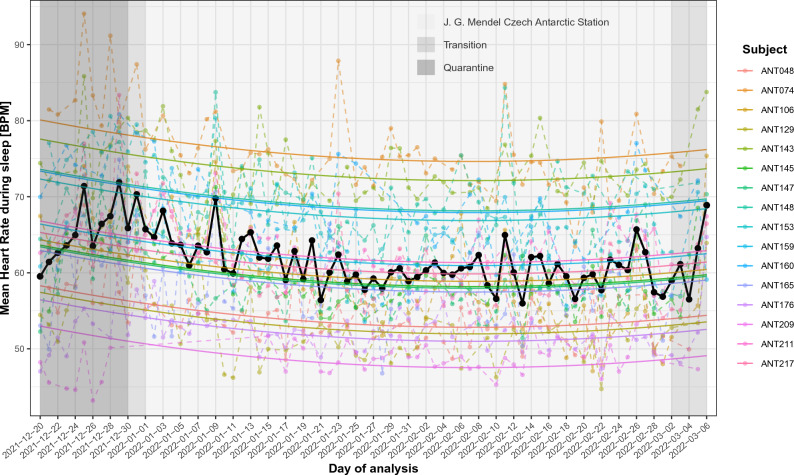
Individual trajectories of mean sleep heart rate during quarantine; transition to J. G. Mendel Czech Antarctic Station, stay at J. G. Mendel Czech Antarctic Station; and transition from J. G. Mendel Czech Antarctic Station assuming quadratic dependence of mean sleep heart rate on time. Black solid line depicts mean values across all participants.

Mean daily sleep heart rate had decreasing tendency as can be seen in Fig. 1. We challenged this observation with statistical analysis using single-factor ANCOVA accounting for heteroskedasticity and correlation between the residuals using generalized least squares (GLS). For each individual, we take into account their own intercept. The mean proportion of missing values by individual was 21.35% (range 7.79% to 51.95%; details in Supplementary Table S4.2)). Results with p-values adjusted using Benjamini–Hochberg correction proved significant influence of time on mean sleep heart rate (β_time_ =  − 0.049, SE = 0.01, p_adj_ = 3.95 × 10^−6^), meaning that with each day, the mean sleep heart rate decreased by 0.049 BPM. Moreover, because the last week’s values have increasing trend, we added time squared into the model. This second model had both statistical coefficients significant, linear, β_linear_ =  − 0.225, SE = 0.04, p_adj_ = 1.0 × 10^−9^, and quadratic, β_quadratic_ = 0.002, SE = 4.46 × 10^−4^, p_adj_ = 1.44 × 10^−6^.

**Figure 2:**
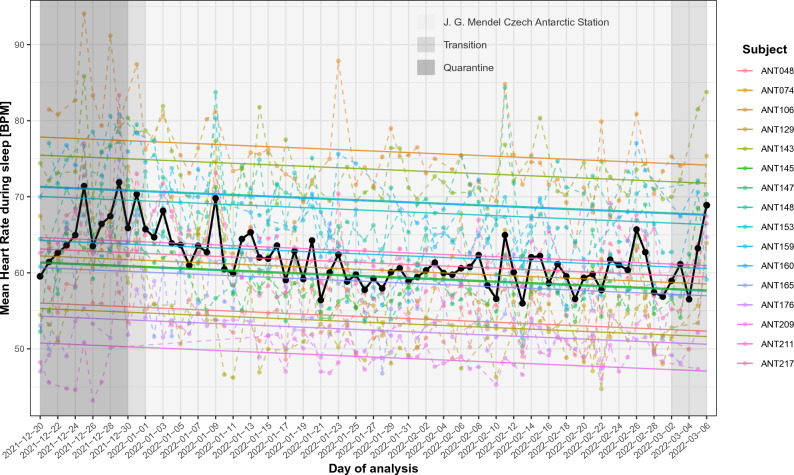
Mean sleep heart rate from stay in J. G. Mendel Czech Antarctic Station. Dashed lines represent real values, full lines are values approximated through modelling. Black depicts mean values of all 16 participants.

If we consider only data from the stay at the station without transition phases (Fig. 2), i.e., 58 days, linear regression coefficient of time on mean sleep heart rate was β_time_ =  − 0.057, SE = 0.009, p_adj_ = 2.00 × 10^−9^. This means, that with each day on the expedition the mean heart rate during sleep decreased by 0.057 BPM. Despite the fluctuation in daily measurements, the trend is still statistically significant and homogenous across all individuals. For this data subset, the mean proportion of missing values was 20.46% (range 9.67% to 51.61%; details in Supplementary Table S4.2)).

#### Mean sleep heart rate and co-factors

We tested relationship between mean sleep heart rate and age, sex, BMI, length of the sleep, and individual’s previous experience with Antarctic. Using the mean sleep heart rate data from the whole expedition, and after adjustment of the p values using Benjamini-Hochberg (FDR) correction, GLS model revealed significant relationship with being a first timer β_first time_ = 16.416, SE = 2.766, p_adj_ = 9.10 × 10^–8^; sleep length β_sleep length_ =  − 0.478, SE = 0.082, p_adj_ = 9.10 × 10^–8^; BMI β_BMI_ = 0.861, SE = 0.298, p_adj_ = 0.03. Meaning that in this model, mean sleep heart rate increased by 16.42 BPM with being a first timer, decreased with each hour of sleep by 0.478 BPM, increased with each kg/m^2^ of BMI by 0.861 BPM. The relationship was not statistically significant with sex, age or with the time spent on expedition. Similar relationship was found also in model considering data from the stay at the J. G. Mendel station only. Here we found significant relationship between being a first timer β_first time_ = 15.739, SE = 2.601, p_adj_ = 6.40 × 10^–8^; length β_sleep length_ =  − 0.395, SE = 0.816, p_adj_ = 2.02 × 10^–5^, time on expedition β_expedition duration_ =—0.395, SE = 0.082, p_adj_ = 2.03 × 10^–5^, but not with sex and age. More details are in supplementary material (Table S6.1, 2).

### Mood trajectory

**Figure 3:**
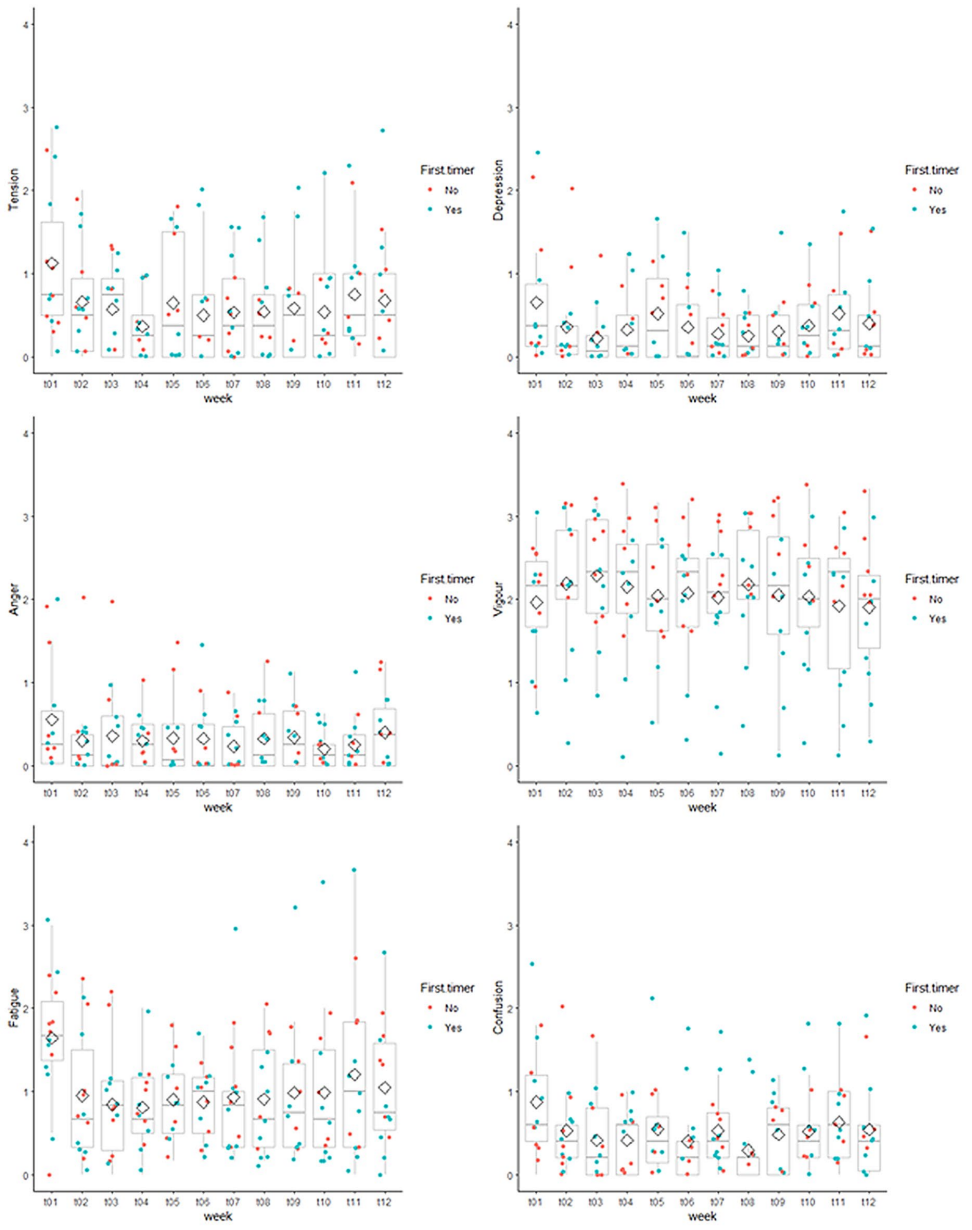
Box plots for week-by-week scores of POMS questionnaire. The mean values are represented by a black dash, median values are represented by a rhomb. First timers’ responses are visualized in green, experienced expeditioners’ responses are in red.

The course of reported mood scores over time is depicted in Fig. 3. Each subscale was subjected to analysis using GLS model which included sex, age, BMI, and previous experience with expedition. Models included intra-subject correlation and heteroskedasticity. They did not find any statistically significant changes in mean values of mood subscales over time. Men reported significantly lower score in Tension, Depression, Vigor, and Confusion subscales compared to women. BMI was significantly positively associated with all negative mood subscales. Age was significantly associated with lower values in Vigor subscale. More details are in supplementary material (Table S7.1–6).

### Mean sleep heart rate and mood

In a complex model with all POMS subscales, time, sex, age, BMI, being a first timer and sleep duration, only association with Vigor subscale was statistically significant (β_vigor_ = − 2.073, SE = 0.576, p_adj_ = 0.012). Being a first timer was borderline significant (β_First-timer_ = 17.915, SE = 6.906, p_adj_ = 0.134). When considering only time spent in Antarctica, statistical significance of the relationship with Vigor diminished, but with being a first timer remained (β_vigor_ = − 1.881, SE = 0.767, p_adj_ = 0.205; β_First-timer_ = 21.238, SE = 6.435, p_adj_ = 0.036). Other relationships were statistically non-significant (Table 1).

**Table 1:** Mean sleep heart rate and mood interrelationships.

Fixed effect	Mean sleep heart rate all expedition			Mean sleep heart rate STATION ONLY		
	Value	SE	p-value adjusted	Value	SE	p-value adjusted
Tension	0.098	0.663	1	− 0.139	0.788	1
Depression	− 0.434	0.848	1	− 0.102	0.849	1
Anger	0.720	0.910	1	1.277	0.955	0.902
Vigor	− 2.073	0.576	0.012	− 1.881	0.767	0.205
Fatigue	0.700	0.485	0.692	0.473	0.541	1
Confusion	− 0.262	0.735	1	− 0.585	0.756	1
time	− 0.264	0.181	0.692	− 0.338	0.214	0.902
Sex (M)	1.844	5.861	1	− 2.275	5.470	1
Age	0.241	0.393	1	0.410	0.329	0.902
FIRST TIMER	17.915	6.906	0.134	21.238	6.435	0.036
BMI	0.548	0.801	1	0.916	0.708	0.902
Sleep duration	− 0.355	0.255	0.692	− 0.135	0.260	1

### Sleep quality trajectory

Although the mean score of subjective sleep quality and sleep latency tended to decrease with time, statistical model did not prove statistically significant difference between weeks. However, several significant associations were found with demographic variables. Men reported worse subjective sleep quality than women, β_sleep_men_ = 0.619, SE = 0.188, p_adj_ = 0.011. Higher BMI was associated with lower subjective sleep quality, β_sleep_BMI_ = 0.121, SE = 0.032, p_adj_ = 0.008. With increasing age, the subjective sleep quality also significantly increased (β_sleep_age_ = − 0.052, SE = 0.015, p_adj_ = 0.011), and sleep latency shortened (β_latency_age_ = − 0.065, SE = 0.015, p_adj_ = 0.0005). In first timers, sleep latency was shorter (β_latency_first.timer_ = − 0.973, SE = 0.319 p_adj_ = 0.023), but effect on sleep quality was not statistically significant. Use of sleep medication was reported by one individual in fourth and fifth week. After that, this individual stopped responding to the questionnaires.

### Mean sleep heart rate and sleep quality

After adjustment of the p-values, the model did not reveal any statistically significant relationship between subjective sleep quality, sleep latency, sleep duration, being a first timer, sex, or age and mean sleep heart rate (Table 2). If we consider only weeks spent in Antarctic station, no statistically significant results were revealed. The strength of model might be decreased due to the high number of missing values (more in S4 Missing values).

**Table 2:** Mean sleep heart rate and sleep quality indicators interrelationships.

Fixed effect	Mean sleep heart rate all expedition			Mean sleep heart rate in station only		
	Value	SE	p-value adjusted	Value	SE	p-value adjusted
Sleep quality	0.158	0.430	1	0.233	0.446	1
Sleep latency	0.958	0.584	1	− 0.187	0.852	1
Time	− 0.233	0.195	1	− 0.322	0.182	1
Sex (male)	0.218	4.163	1	3.544	8.448	1
Age	0.369	0.362	1	− 0.331	0.568	1
First timer	19.322	7.530	0.293	10.830	10.614	1
BMI	0.963	1.011	1	0.875	1.251	1
Sleep duration	− 0.085	0.276	1	0.281	0.232	1

### Perceived stress trajectory

Evaluation of the mean values and variance depicted in the graph (S11.1) indicates decrease of perceived helplessness from first to seventh week with small subsequent increase. However, a simple model with time did not reveal any statistically significant differences between weeks. In a complex model with co-factors, men reported lower values than women with borderline statistical significance, β_men_ = − 0.923, SE = 0.323, p_adj_ = 0.06. Also, higher BMI was significantly related to higher scores, β_BMI_ = 0.159, SE = 0.050, p_adj_ = 0.043.

The perceived lack of self-efficacy was relatively stable within the expedition time, but with considerable variation. There were three apparent drops in mean values during the expedition, namely in fifth to sixth week, eighth and tenth week. This coincided with periods of preparations for a camp period and transits from Antarctica (Table 4). However, models with adjusted p values by Benjamini-Hochberg (FDR) correction did not reveal statistically significant differences between weeks.

The mean total score was decreasing from first to seventh week with two peaks in fourth and sixth week. From the seventh week it was continuously increasing. GLS model again did not revealed significant differences between weeks. Men reported lower values, β_men_ = − 8.419, SE = 2.936, p_adj_ = 0.061, and higher BMI was borderline significantly related to higher scores, β_BMI_ = 1.360, SE = 0.451, p_adj_ = 0.061. More details are in Supplementary Table S11.1.

### Mean sleep heart rate and perceived stress

The model did not reveal any statistically significant relationship between perceived helplessness, lack of self efficacy and mean sleep heart rate during the whole expedition, nor at the station (Table 3). Similarly, as with subjective sleep quality, the strength of model might be decreased due to the high number of missing values (more in S4 Missing values). After adjustment of p-value by Benjamini–Hochberg correction, quadratic time (β_time_ = − 3.017, SE = 0.795, p_adj_ = 0.006) and being a first timer (β_first.timer_ = 18.849, SE = 6.439, p_adj_ = 0.037) was significantly related to mean sleep heart rate during the stay at the station (Table 3).

**Table 3:** Mean sleep heart rate and perceived stress interrelationship.

Fixed effect	Mean sleep heart rate all expedition			Mean sleep heart rate in station only		
	Value	SE	p-value	Value	SE	p-value adjusted
Perceived helplessness	− 0.685	0.505	0.889	0.081	0.547	1
Lack of self efficacy	0.822	0.409	0.585	0.103	0.491	1
Time	− 0.254	0.152	0.615	− 3.017	0.795	0.0056
Sex (male)	0.981	6.479	1	0.490	5.287	1
Age	0.114	0.423	1	0.280	0.324	1
First timer	16.919	8.022	0.686	18.849	6.439	0.037
BMI	0.794	0.875	1	0.844	0.734	1
duration	− 0.490	0.271	0.601	− 0.077	0.234	1

## Discussion

This study analyzed trajectories of stress reaction indicators proxied from mean sleep heart rate, emotions, perceived stress, and indicators of sleep problems in a summer expedition to a small and isolated Antarctic station, as well as preceding COVID-19-related quarantine before the entry to Antarctica. The findings revealed that the mean sleep heart rate was increasing during the quarantine and the transportation phases, indicating a higher stress. By contrast, during the stay at the Antarctic station, the mean sleep heart rate decreased by up to 1.71 BPM per 30 days, suggesting a restorative effect and reduced sympathetic nervous system activity during sleep. These results differ from previous studies that focused on daytime heart rate and found no significant changes^5,29,30^ or even increases^32^. This might be possibly caused by their lower sampling rates, general differences in cold adaptation among participants with various geographical origin^44,45^, or contextual differences such as in the workload, social, practical aspects, etc. More interestingly, results from GLS models suggest that previous experience have an influence on the decrease in mean sleep heart rate over time. This provides evidence for results of previous research which recognize experience as an important factor of adaptability to challenging environment^12^. The influence of positive emotions on mean sleep heart rate was found to be statistically significant only in the model considering data from the entire expedition, not in the model focused solely on the stay at the J. G. Mendel station. This suggests the potential significance of positive emotions in promoting restfulness during challenging situations, such as the transition phases experienced in this expedition, as evidenced by the increase in mean sleep heart rate. These findings align with prior research that has reported a similar association between positive emotions and stress indicators^35,39,46,47^. On the other hand, we were not able to find statistically significant relationship with stress and self-efficacy which have been found in previous study^48^. Age did not have a significant effect on the mean heart rate decrease, which might be caused by its association with previous experience that is usually present in older individuals. None of the sleep-related variables, negative emotions, or BMI revealed a statistically significant relationship with the mean sleep heart rate.

Regarding the psychological indicators of the stress, the aspection of graphs indicates a considerable decrease in reported negative emotions and perceived stress, particularly in the perceived helplessness subscale. These results support previous findings on salutogenic effect of summer Antarctic expeditions^9^. However, weekly differences in mean scores for mood subscales, perceived stress, or subjective sleep quality did not reach statistical significance. Tendency for decreasing sleep quality and increasing sleep latency aligns with previous studies^9,49^. GLS models revealed that men reported worse sleep quality which is somewhat contrary to studies on sleep quality in general population which usually finds worse sleep quality in women^50^. On the other hand, men reported lower values in perceived stress, and all mood subscales. First-timers reported significantly higher values of perceived helplessness, and depressive symptoms, but after adjustment of p-values for multiple hypotheses testing, this association diminished. Age was related to higher Vigor, higher subjective sleep quality, and shorter sleep latency. Lastly individuals with higher BMI reported lower sleep quality, perceived stress, and higher values in all negative mood subscales but not the positive ones.

Our study breaks new ground in the field of ICE studies by integrating longitudinal daily monitoring of physiological markers using wearables with weekly self-report assessments on psychological well-being, and sleep quality using subjective and objective metrics. The results demonstrate the feasibility and advantages of our study design, which overcomes the limitations of small sample sizes typically found in this field. Unlike previous studies that employed sampling intervals longer than week^5^, month^30,32,36^ or even more sporadic^29,35^. Moreover, these studies usually measured heart rate during the daytime^30,36^. Nighttime measurements are more reproducible, less impacted by other daily stressors, and previously determined as the best timeframe for resting heart rate measurement^51^ with the highest clinically predictive value^52^. Next, previous studies did not compare physiological data with emotions, perceived stress, or sleep quality^29,30,32^ on both objective (sleep length and heart rate measured by a sporttester) and subjective (perceived sleep quality and latency measured by PSQI) level^53^. Replicating this study design in various expeditions and missions within isolated, confined, extreme, and controlled environments hold great potential for advancing our understanding of individual adaptation and identifying crucial protective factors.

Our study advances understanding on physiological, psychological and demographical factors that promote positive aspects of living and working in ICE conditions^9^. This knowledge is critical for universities, research organizations, and expedition planners involved in preparing individuals for expeditions in extreme environment^9,13^. The insights gained from our study are also applicable to teams operating in extreme action settings, such as military, medical, police, and fire personnel, as well as aviation and naval crews and control room operators^13^. Moreover, our results may be informative for expedition planners in charge of logistics, and national operators that will be developing protocols and mitigation strategies for preventing pandemic spread in Antarctica, particularly in the context of the recent COVID-19 pandemics^54^. By identifying the potential stress impact of transportation phases and short-term preventative measures, our study contributes to the development of effective mitigation strategies. Furthermore, the implications of our research extend beyond specialized environments. The insights gained are informative for the general public affected by global crises, such as climate change-induced hostile environments^55^ or individuals experiencing protective isolation measures during COVID-19-related^56,57^. Lastly, our results are relevant for long-duration spaceflight missions (LDSM). The challenges faced in ICE environments parallel many aspects of LDSM, such as the limited medical care and no option to be evacuated or rescued quickly. The resupply is infrequent, and communication with outer world limited with the data bandwidth and power constrains^8^. There sensory stimulation is low and recreation options minimal. The aspects of physical and social isolation are high. The team is highly autonomous, workload and work are moderate to high in danger, and the tasks that environmental scientists and technicians on expedition perform are somewhat similar to what astronauts will undertake in future LDSM. While previous space-analogue research in Antarctica has primarily focused on winter-overs for their resemblance to spacecraft travel. However, summer expeditions in Antarctica may be more appropriate as an analogue to Lunar missions. The prolonged Lunar day, with a length of 14.5 Earth days, and the environmental science fieldwork conducted by researchers in Antarctica align closely with the challenges and activities that astronauts will encounter on Lunar missions. Therefore, our study provides valuable insights for the preparation, well-being, and success of future astronauts in LDSMs, supporting the exploration and habitation of space by humankind.

This study is subject to several limitations inherent to studies in ICE, which should be addressed. Firstly, the small sample size may reduce the statistical power. This means that some true but small effects may not reach statistical significance. Moreover, the coefficients should be considered with caution^58–60^. To mitigate this concern, we implemented statistical analysis that accounted for fixed effects, and optimized models to control for autocorrelation and heteroscedasticity, thus controlling the Type I error. Such model was used for testing the importance of other variables as was previously recommended^60,61^. It is also worth noting that some results may have been influenced by missing data. Furthermore, the generalizability of our findings may be limited by the non-random selection of individuals participating in Antarctic expeditions, as those who choose to visit Antarctica are usually not typical population, and repeat visitors often enjoyed prior expedition experience. As a result, the applicability of our results may not extend to all individuals in general population. Lastly, our heart rate measurements only provided point estimates of mean values and did not allow for the computation of heart rate variability, which could have provided additional valuable insights.

Future studies should aim to replicate our study design to get larger datasets, as it has demonstrated feasibility and provided valuable insights, particularly in contexts where teams operate in isolated geographical locations or space analogs^13^. Additionally, incorporating continuous physiological signal data collection in future studies would significantly enhance the scope of data analysis and further deepen our understanding. Furthermore, it is crucial for future studies to monitor expedition crews both before departure and upon their return to civilized society, in order to examine the enduring effects of expeditions and the process of re-adaptation.

## Materials and methods

### Participants

#### Demographic and related variables

Studied population consisted of 16 Antarctic expeditioners (5 women; mean age 35.41, SD 10.51). Twelve individuals were Czech, three were Slovaks permanently residing and working in the Czech Republic, one participant was British. Regarding education levels achieved, nine individuals were university graduates, four individuals were post-graduates, two individuals finished high school and one elementary school. Half of the crew attended the expedition for the first time, the other half had previous experience. Description of individuals is in the Supplementary Table S1.

#### Anthropometric data

The mean body mass index of individuals was 24.18 (SD 3.30) kg/m^2^ for men, and 21.93 (SD 3.04) kg/m^2^ for women. Wrist circumference was measured at the level of the tip of the styloid process of the radius and ulna, with outstretch of the hand using. Men had mean circumference of 17.91 (SD 1.38) cm, and women of 16.06 (SD 1.10) cm. Skin tone was assessed using Fitzpatrick scale^62^. All participants felt under 1^st^ or 2^nd^ degree of the scale. No participant had tattoo on the wrist area. The used scale and photographs of wrist areas from those who agreed with it are in Supplementary section S3.

#### Medical data

During the first measurement, one individual reported use of cardiac medication, another use of asthma and allergy medication, and other the use of medication for thyroid hormone deficiency. One individual reported use of anticoagulant medication during the flight. During expedition, use of sleeping pills was reported in one individual. More information is in the Supplementary Table S2.

### Study design protocol

This study is part of a longitudinal study on the trajectory of stress conducted during the summer Antarctic expedition of 2021/2022 organized by the Czech Antarctic Research Programme based at the Masaryk University. Ethics approval was obtained from Masaryk University Ethics Committee and all methods were performed in accordance with the relevant guidelines and regulations. The expedition took place at the Johann Gregor Mendel Czech Antarctic Station, located on James Ross Island (63°48′02″ S, 57 52′54″ W, altitude 10 m). The expedition began with departure from the Czech Republic on December 16, 2021. The transportation period from the Czech Republic to Chile lasted three days. Due to Covid-19 related restrictions and a mandatory 10-day quarantine, the expedition team was isolated in a hotel in the city of Punta Arenas starting from December 18. In the measured period, there was 16:08 to 15:48 h:m of daylight between 20 and 26th of December, compared to Czechia where was 8:12–8:13 h:m of daylight on 20th to 26th of December^63^. On 30th of December, the team flew to King George Island and was then transported by boat to James Ross Island, arriving on 31st of December, where they were stationed at the J. G. Mendel Czech Antarctic Station until March 2. On 1^st^ of January 2022 there was 20:47 h:m of daylight, on 2nd March it was 14:14 h:m. This is considerably alternated compared to conditions in Czechia where on 1st of January was 8:17 h:m of daylight, on 2nd March it was 11:03 h:m^63^. The team was then transported from James Ross Island to King George Island and accommodated at the General Artigas Uruguayan scientific station until 6th of March. The expedition concluded with the team's arrival back in the Czech Republic or the United Kingdom on 8th of March, 2022.

Potential participants were recruited during the pre-expedition meeting in November 2021. Recruitment of expeditioners was conducted by the Czech Antarctic Research Program (CARP). To these selected and confirmed participants, we presented the research idea, study plan and explained potential benefits and risks. After that, participants were given informed consent for signature. Their signature and voluntary willingness to participate was the inclusion criterion for the study. Because expeditioners were subjected to medical check-up, the only exclusion criterion in the present study was a withdrawal of the consent.

### Heart rate measurement

#### Technology specifications

The design and protocol for collection of heart rate data using wrist wearables is based on the standardized guidelines provided by Nelson et al.^14^ and is reported in S1 and S2 tables. We used Garmin 55 Forerunner (version 4.11 [DFU-5beea5] or 3.03 [DFU-5beea5]). No update on software was performed during the study. The device uses the Garmin Elevate V3 optical sensor with an Activity Tracking mode that samples in 1 Hz interval. The validity of wearables using Garmin Elevate V3 sensor (specifically Forerunner 235^64,65^; and Vivosmart HR + ^66^) during rest had acceptable agreement with ECG (r = 0.88)^64^ and Polar RS400 chest strap (r = 0.99)^65^. Reliability of heart rate measured by Garmin Vivosmart HR + was reported previously for walking test and household activities and ranged from moderate to excellent^66^. Pre-processing of the data is described in the subsection Data analysis below.

#### Participants characteristics

Thirteen participants (81.25%) wore smart watches on their non-dominant hand, only three reported wearing watches on dominant hand. Six individuals reported wearing smart watches tightly, another six reported wearing smart watches in middle tightness, four individuals reported wearing them loosely. Characteristics are detailed in Supplementary S2 Potential covariates for Heart Rate.

#### Instructions for participants

Before wearing the watches, participants received instructions in a pre-expedition manual. Instructions on how to wear watches properly were derived from the manual Garmin provides. Participants were informed about the importance of wearing the watches correctly for accurate data collection. They were advised to ensure that the watches remained in close contact with the skin without causing any discomfort or constriction. Considering the natural changes in arm size throughout the day, participants were instructed to adjust the strap as necessary. Additionally, participants were advised to maintain wrist hygiene and clean the watches with pure water when required. Detailed instructions can be found in Supplementary section S2.

### Questionnaires

A demographic questionnaire was delivered to participants after they signed informed consent, before departure to the expedition. This study uses questions about the sex, age, nationality, highest achieved education, height, weight, and previous experience with Antarctic expedition (e.g., are you on this expedition for the first time, or have you been one or more times before).

Mood was assessed using the Czech version of a shortened version of the Profile of Mood States (POMS-SF)^67^. A 37-item self-assessment inventory evaluating mood on a 5-item scale ranging from “not at all” to “extremely”. The items were then summed according to the manual, resulting in six factors: tension-anxiety, depression-dejection, anger-hostility, vigor-activity, fatigue-inertia, confusion-bewilderment. Because each factor has different item loading, final scores were standardized to range from 0 to 4. Although the Czech version of the subscales had different item loadings compared to the original scale^68^, we opted to use the original loadings due to their better internal consistency in our sample. More information on both methods of calculation can be found in the supplementary materials.

Perceived stress was evaluated using the Czech version of 10-item Perceived Stress Scale (PSS-10). The questions are ranked on the 4-point Likert-like scale and address how unpredictable, uncontrollable, and overloaded individual feels. The range of possible answers is 0 to 40. Answers are further divided into Perceived Helplessness and Lack of Self Efficacy subscales, each ranging from 0 to 4. Validity and reliability of the Czech version has been previously evaluated and reported as satisfactory^69^.

Subjective sleep quality, Sleep latency and Use of sleep medication was measured using two questions from Pittsburgh Sleep Quality Index (PSQI). PSQI is well established questionnaire in the field of sleep research^70,71^. However, for the purpose of this study shortening of the inventory was required, we chose only questions concerning concept of highest interest. All three subscales have a range from 0 to 3.

### Data analysis

Data were downloaded from Garmin watches through weekly back-ups using USB cable. The data were in. FIT format and we used the FITfileR package^72^ from R software version 4.1.1. to analyze them. Data were manually controlled and cleared from significantly higher heart rate during beginning and end of the sleep phase, indicating awake time. If there were two recordings for one day (e.g., one beginning at 0:30 and other at 23:45), the latter was considered as belonging to the following day. In rare cases, some recordings had to be excluded from the analysis, such as recordings of heart rate during naps, recordings longer than 20 h, or short recordings with mean value above 100 BPM. Moreover, some days also had missing data (approx. one day per week). After elimination of data unsuitable for analysis, mean, minimal and maximal heart rate during sleep, variance and length of the sleep were calculated for each recording from available data for each day.

Based on the recordings, two datasets for heart rate analysis were constructed. The first dataset consisted of individual daily recordings from 20^th^ December 2021 to 6^th^ March 2022 (Table 4). We chose to omit days before 20^th^ because of better weekly division and because first days had higher rate of missing data. For each day, above-described summary statistics were calculated. The second dataset was based on mean heart rate values based on the weeks from first dataset. Every week begins with Monday and ends with Sunday according to the division detailed in Table 4.

**Table 4:** Weeks and their description.

Week	Date range	Expedition phase
1	20.12.–26.12. 2021	Quarantine in Punta Arenas, Chile
2	27.12–31.12. 2021	Transit to Antarctica
3	2.1.–9.1. 2022	Stay at J. G. Mendel Czech Antarctic Station
4	10.1.–16.1. 2022	Stay at J. G. Mendel Czech Antarctic Station
5	17.1.–23.1. 2022	Stay at J. G. Mendel Czech Antarctic Station
6	24.1.–30.1. 2022	Stay at J. G. Mendel Czech Antarctic Station
7	31.1.–6.2. 2022	Stay at J. G. Mendel Czech Antarctic Station (first camp period 1.-9.2.)
8	7.2.–13.2. 2022	Stay at J. G. Mendel Czech Antarctic Station
9	14.2.–20.2. 2022	Stay at J. G. Mendel Czech Antarctic Station (second camp period 14.-18.2.)
10	21.2.–27.2. 2022	Stay at J. G. Mendel Czech Antarctic Station
11	28.2.–6.3. 2022	Transit from Antarctica

To analyse trajectories of mean sleep heart rate, time series from each of the 16 participants were subjected to single-factor ANCOVA accounting for correlation between the residuals from the same participant and also for heterogeneity of variances between the participants using generalized least squares. The time was a numeric and ID a categorical covariate, without interaction. Next, data were subjected to linear modelling using generalized least squares through gls () function in nlme R package^73^. Models considered intra-subject correlations but not inter-subject correlations. Several models were calculated and compared using ANOVA likelihood ratio test or by examining the ACF for models’ residual. According to the Autocorrelation Function (ACF) and Partial Autocorrelation Function (PACF) for individual time series of each subject, the correlation structure in the model was selected. Based on these comparisons, model with heteroscedasticity assumption and autoregressive correlation structure of order two (AR2) was selected for further analyses. Results from the analysis are interpreted with the estimate of coefficient beta (β) and standard error (SE) of the estimate. All tests were performed in a two-tailed, with significance level alpha set to 0.05. Assumption of normality was verified by graphical methods (histogram with curve of normal distribution) and by the Shapiro–Wilk test of normality. The independence of residuals in the model was verified by ACF.

### Supplementary Information

Supplementary Information.

## Data Availability

The datasets generated during and/or analyzed during the current study are not publicly available due to ethical consideration and participants protection but are available from the corresponding author on reasonable request.
