# Supplementary material for: Physiological evidence of stress reduction during a summer Antarctic expedition with a significant influence of previous experience and vigor
Source: Sci Rep. 2024 Feb 17;14:3981. doi: 10.1038/s41598-024-54203-9 (PMC10874375; doi:10.1038/s41598-024-54203-9)
Supplement: Supplementary file 1 — Supplementary Information. [file 41598_2024_54203_MOESM1_ESM.pdf]

Temporal changes in stress during a summer Antarctic expedition: physiological proof of salutogenesis, especially along with experience, vigor, and self-efficacy

Lucie Ráčková *Department of Environmental Physiology, RECETOX, Faculty of Science, Masaryk University, Kotlarska 2, Brno, Czech Republic*
Tomáš Pompa *Research Centre for Toxic Compounds in the Environment (RECETOX), Masaryk University, Brno, Czech Republic*
Filip Zlámal *Department of Physical Activities and Health Sciences - Faculty of Sports Studies, Masaryk University, Brno, Czech Republic*
Miloš Barták *Department of Experimental Biology, Masaryk University, Kamenice 5, 625 00 Brno, Czech Republic*
Daniel Nývlt *Department of Geography, Masaryk University, Brno, Czech Republic*
Julie Bienertová-Vašků *Department of Food Technology, Mendel University, Brno, Czech Republic*

This document provides supplementary materials for the main article, including diagnostics graphs, tests of normality, whole models outputs etc. We also provide for some models used in the main article a short discussion about why we chose that particular model and a comparison with similar models under different assumptions. Document was written in RMarkdown.

Contents

S1 Demographics & anthropometry **S2**

S2 Potential covariates for Heart Rate **S4**

S3 Skin scale **S5**

S4 Missing values **S9**

S5 Mean sleep heart rate trajectory **S10**
S5.1 All expedition S10
S5.1.1 Linear trend S10
S5.1.2 Quadratic trend S12
S5.2 Station only S16

S6 Mean sleep heart rate and co-factors **S19**
S6.1 All expedition S19
S6.2 Station only S20

S7 Mood trajectory **S23**
S7.1 Tension S23
S7.2 Depression S24
S7.3 Anger S25
S7.4 Vigour S26
S7.5 Fatigue S27
S7.6 Confusion S28

S8 Mean sleep heart rate and mood **S29**
S8.1 All expedition S29
S8.2 Station only S31

S9 Sleep quality trajectory **S33**
S9.1 Subjective sleep quality S33
S9.2 Sleep latency S34

S10 Mean sleep heart rate and sleep quality **S34**
S10.1 All expedition S34
S10.2 Station only S36

S11 Perceived stress trajectory **S39**
S11.1 Perceived helplessness S39
S11.1.1 Simple model with time S39
S11.1.2 Complex model with co-factors S39
S11.2 Lack of self efficacy S40
S11.2.1 Simple model with time S40
S11.2.2 Complex model with co-factors S40
S11.3 Mean total score S41
S11.3.1 Simple model with time S41
S11.3.2 Complex model with co-factors S41

S12 Mean sleep heart rate and perceived stress **S42**
S12.1 All expedition S42
S12.2 Station only S44

S13 P-value adjustment **S46**

S1 Demographics & anthropometry

Firstly, we focus on descriptive statistics of participant information, such as demographic and anthropometric data. We begin with categorical demographic data – Sex, Nationality, Academic rank and First timer. For our purposes, only Sex and First timer are considered as covariates in further models.

Table S1.1: Simple summary statistics of Sex variable. Absolute and relative numbers of observations are reported. There are no missing values.

	Number of observations	Percentage of observations
Female	5	31.25
Male	11	68.75

Table S1.2: Simple summary statistics of Nationality variable. Absolute and relative numbers of observations are reported. There are no missing values.

	Number of observations	Percentage of observations
British	1	6.25
Czech	12	75
Slovak	3	18.75

Table S1.3: Simple summary statistics of Academic rank variable. Absolute and relative numbers of observations are reported. There are no missing values.

	Number of obs.	Percentage of obs.
Elementary	1	6.25
High school	2	12.5
University Graduate	9	56.25
University Postgraduate	4	25

Table S1.4: Simple summary statistics of First timer variable. Absolute and relative numbers of observations are reported. There are no missing values.

	Number of observations	Percentage of observations
No	8	50
Yes	8	50

Now we consider only variable Age. In table below (Table S1.5) descriptive statistics of age for different subgroups are shown.

We can also perform Welch Two Sample t-test of age between two groups of participants (Table S1.6). We can see that First timers are significantly younger than more experienced participants (p-value is equal to 0.004).

In two tables below anthropometric data for men (Table S1.7) and women (Table S1.8) separately are shown. We report participant's height, weight, BMI, and circumference (Processus styloideus radii and The narrowest area). For more detailed information see Table 2 mHealth wearable heart rate metadata checklist 1: descriptive reporting of sample. (nature.com) and Table 3 mHealth wearable heart rate metadata checklist 2: potential covariates. (nature.com).

Table S1.5: Descriptive statistics of age in the whole sample, men or Women only, and first timers or experienced expeditioners.

	Ntotal	Nmiss	Mean	SD	SE	Median	Min	Max
Age	16	1	35.41	10.51	2.71	33.08	24.17	57.67
Sex - Male	11	1	38.28	11.45	3.62	34.5	24.17	57.67
Sex - Female	5	0	29.65	5.41	2.42	26.42	25.42	37.58
First timer - No	8	1	43.63	9.48	3.58	44.25	33	57.67
First timer - Yes	8	0	28.21	4.1	1.45	26.21	24.17	35

Table S1.6: Welch Two Sample t-tests of differences in age inbetween sex and experience categories. First three columns correspond to Males / Experienced expeditioners, another three columns correspond to Females / First timers. Then value of test statistics t, degrees of freedom and p-value are reported alongside with effect sizes using Cohen's d.

	n1	Mean1	SD1	n2	Mean2	SD2	t	df	p.value	d
Sex	10	38.283	11.449	5	29.650	5.411	1.983	13	0.069	0.864
Firts timer	7	43.631	9.480	8	28.208	4.104	3.989	7.946	0.004	2.169

Table S1.7: Anthropometric data for men (n = 11) separately. The mean, standard deviation, minimum and maximum are shown for these variables.

	Mean	SD	Min	Max
Height [cm]	180.2727	10.0209	159	190
Weight [kg]	78.9091	14.8826	57	112
BMI [kg/mˆ2]	24.1764	3.2989	20.5300	31.3500
Circumference (Processus styloideus radii) [cm]	17.9045	1.3779	15.3000	19.8500
Circumference (The narrowest area) [cm]	18.0091	1.5378	15	20.5000

Table S1.8: Anthropometric data for women (n = 5) separately. The mean, standard deviation, minimum and maximum are shown for these variables.

	Mean	SD	Min	Max
Height [cm]	167.4000	6.2290	163	178
Weight [kg]	61.8000	11.8195	47	79
BMI [kg/mˆ2]	21.9340	3.0384	17.6900	24.9300
Circumference (Processus styloideus radii) [cm]	16.0600	1.0997	14.1500	16.8000
Circumference (The narrowest area) [cm]	16.2400	0.8591	14.8000	16.9000

S2 Potential covariates for Heart Rate

In this short section we examine two potential covariates for modelling mean heart rate during sleep – Wrist placement and Tightness[1].

Wrist placement

Three participants reported wearing the heart rate monitor on their dominant hand (18.75 %), 13 reported wearing them on their non-dominant hand (81.25 %). One participant reported being ambidextrous, one other reported changing placement if skin irritation emerged.

Tightness

Six participants (37.5 %) wore the heart rate monitor tight, other six reported wearing them of middle tightness and four (25 %) wore them loose.

Naturalistic use by participants vs explicit instructions by experimenters

Participants received explicit instructions by experimenter, the instructions are below. Following them was responsibility of the participants.

The data are well only if you wear the watches well. The hear rate is measured using an optical sensor which detects a minimal change in blood flow on the wrist. To get the best estimate of your heart rate and its variability, you need to do this:

- The watches must be in close contact with the skin. There must not be a gap and they must not slide on the hand.
- The watches must not strangle your hand, they must be comfortable.
- The correct tightening can be checked as follows: the movement of the watches corresponds with the movement of the skin. The optical sensor is still on its place even if the hand moves.
- During the day, adjust the strap as needed.
  - We might be able to get a textile straps instead of the silicone ones, but it can not be guaranteed.
- While showering, please clean your wrist properly. Ideally clean the watches using the water.
  - Creams etc. can be cleaned using alcohol based cleaning solution (for this purpose I bring 70% IPA on the expedition).

For more details see www8.garmin.com.

Medication

Type of medication	Number of participant
Cardiovascular medication	1
Anticoagulants for flight	1
Asthma and allergy medication	1
Hypothyroidism medication	1

[1] for more details see this table.

S3 Skin scale

In this section we focus on Skin tone rated against Fitzpatrick Skin typing scale. Skin tone affects the reflectance and could distort our data. We include this section on the recommendation of this article[2].

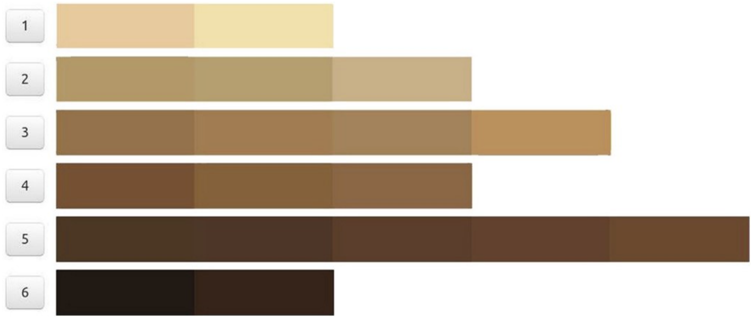

Figure S3.1: Source of the picture: Gupta, V. & Sharma, V. K. Skin typing: Fitzpatrick grading and others. Clin. Dermatol. 37, 430–436 (2019).

Pictures of hands were taken between 18.1. and 21.1. 2022. We took a picture of hand on which the watches were worn, if participant agreed with it. Photography of hand with watches was taken too when possible.

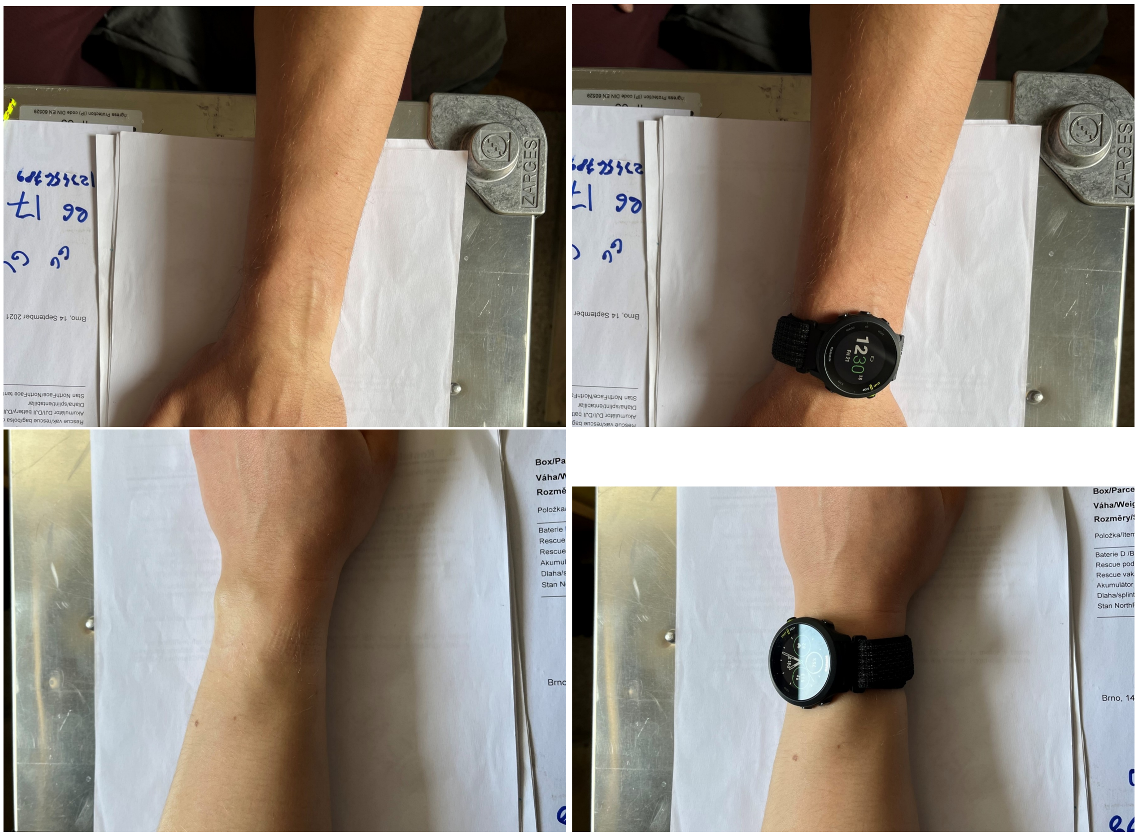

[2]Nelson, B.W., Low, C.A., Jacobson, N. et al. Guidelines for wrist-worn consumer wearable assessment of heart rate in biobehavioral research. npj Digit. Med. 3, 90 (2020). https://doi.org/10.1038/s41746-020-0297-4

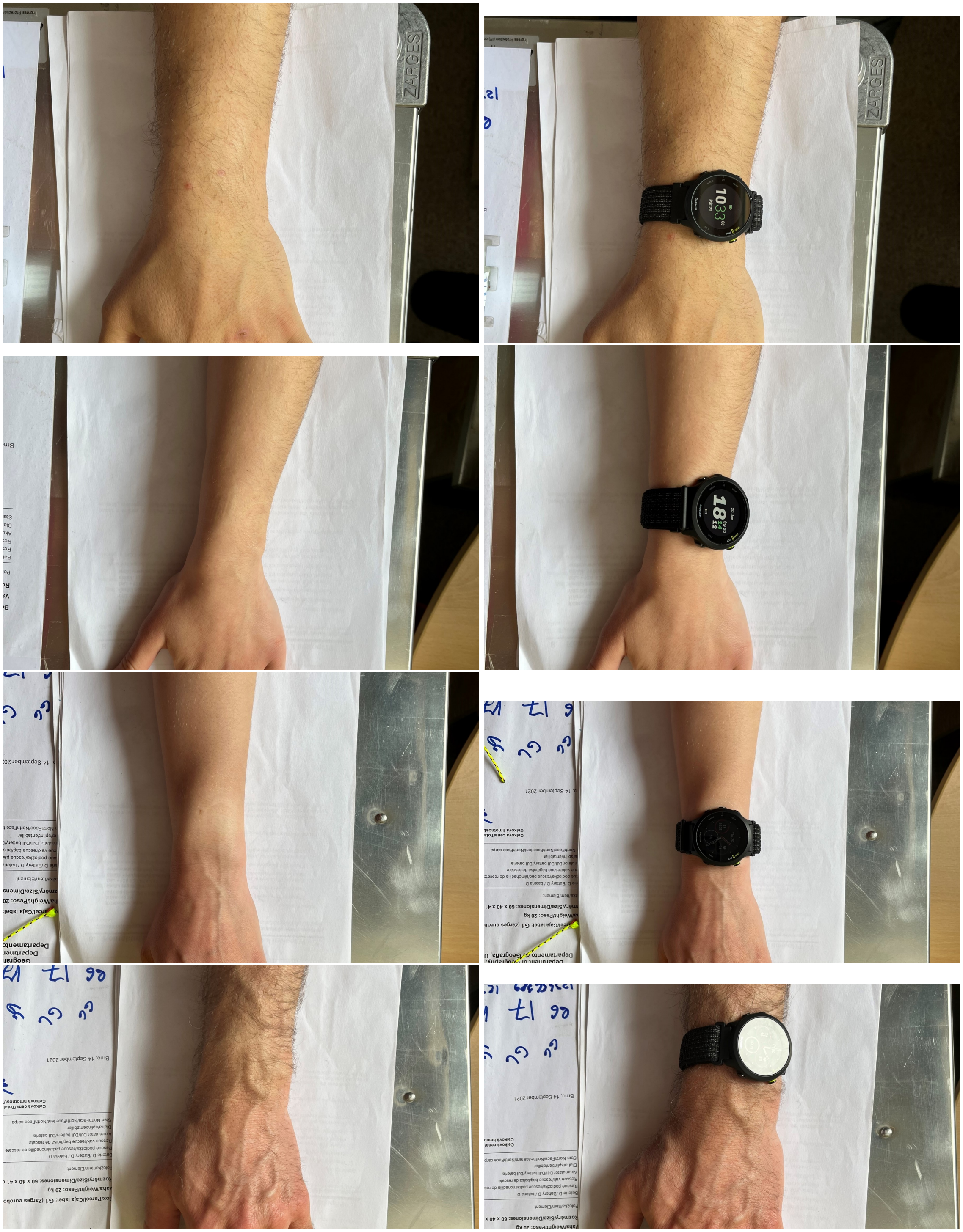

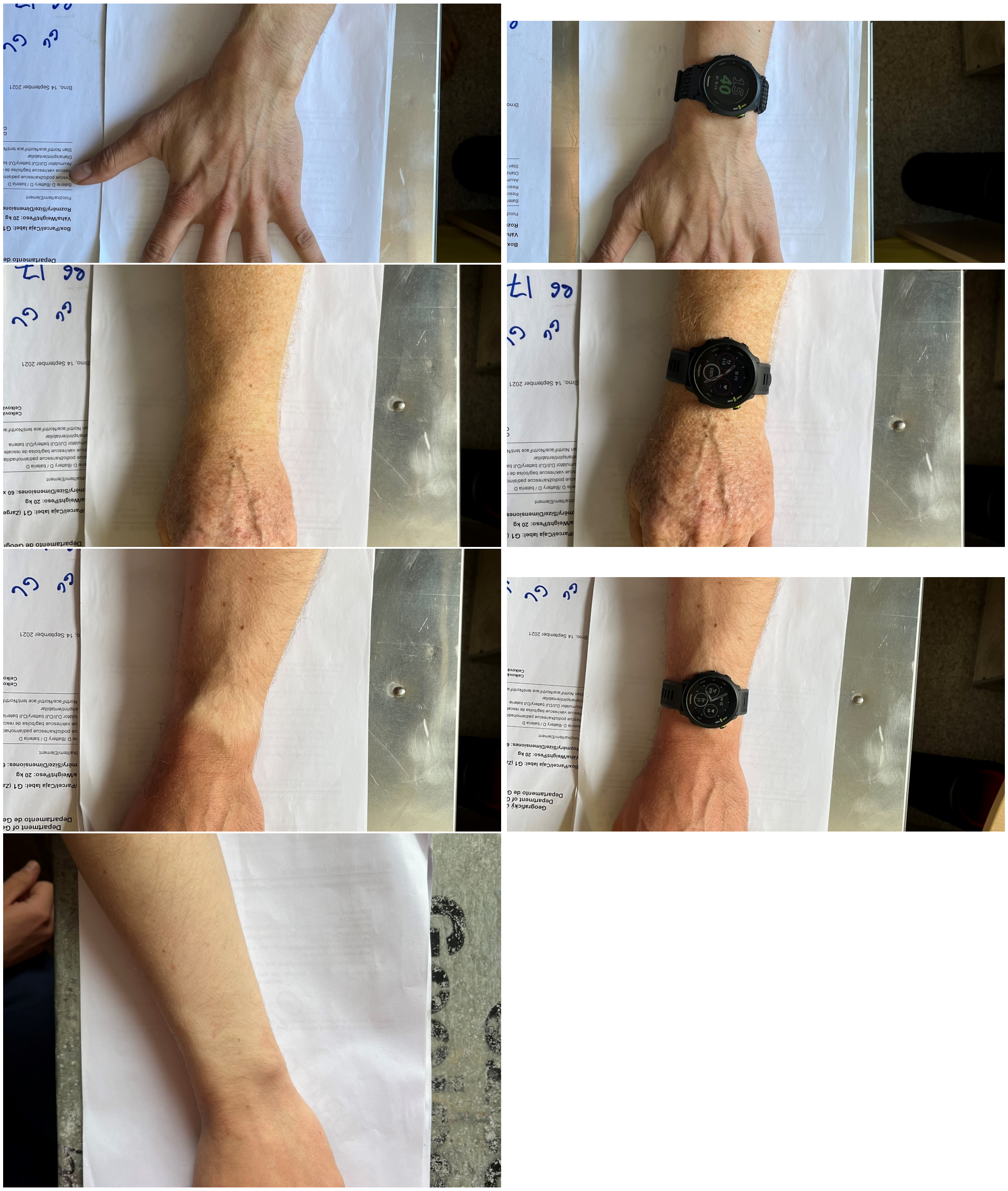

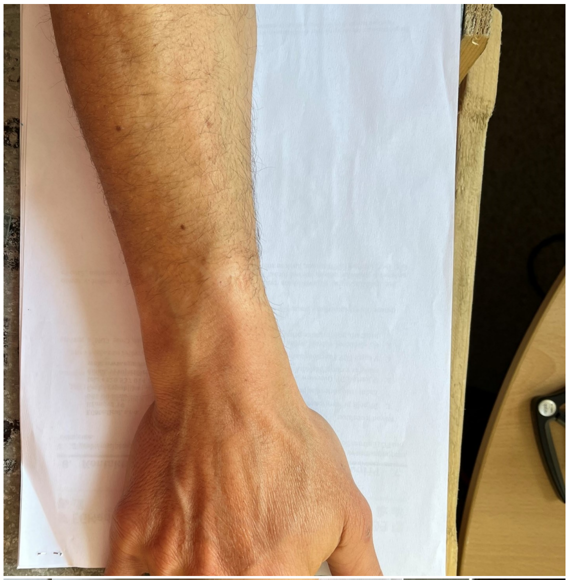
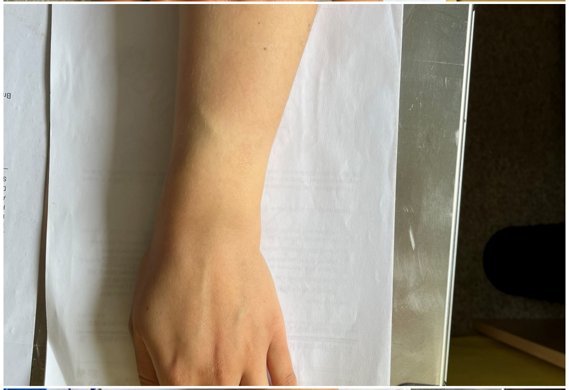
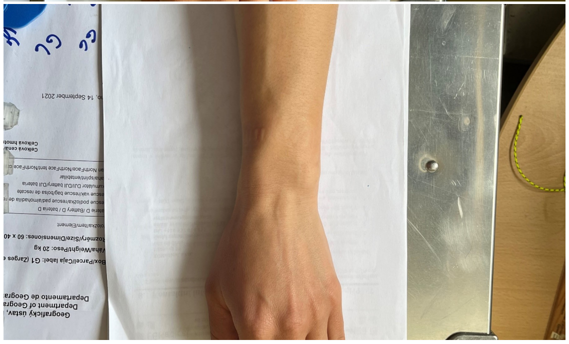

S4 Missing values

Table S4.1: Table of missing daily recordings of heart rate during sleep. The first two columns correspond to all expedition, the last two columns correspond to J. G. Mendel Czech Antarctic Station only. Firstly, the absolute numbers of missing values for each subject are reported, then the proportions of missing values for each subject are calculated.

ID	#NA's - all	Proportion NA - all	#NA's - station	Proportion NA - station
ANT048	16	0.208	13	0.210
ANT074	15	0.195	9	0.145
ANT106	8	0.104	6	0.097
ANT129	19	0.247	14	0.226
ANT143	14	0.182	12	0.194
ANT145	20	0.260	13	0.210
ANT147	6	0.078	6	0.097
ANT148	19	0.247	16	0.258
ANT153	14	0.182	8	0.129
ANT159	21	0.273	19	0.306
ANT160	40	0.519	32	0.516
ANT165	11	0.143	9	0.145
ANT176	11	0.143	9	0.145
ANT209	27	0.351	21	0.339
ANT211	11	0.143	8	0.129
ANT217	11	0.143	8	0.129

Table S4.2: Summary statistics of numbers of missing daily recordings of heart rate during sleep. The first two rows correspond to all expedition, the last two rows correspond to J. G. Mendel Czech Antarctic Station only. First, the number of participants is reported, then the mean, standard deviation, minimum and maximum of absolute number and proportion of missing values for one subject are calculated.

Statistic	N	Mean	St. Dev.	Min	Max
#NA's - all	16	16.43750	8.27018	6	40
Proportion NA - all	16	0.21347	0.10740	0.07792	0.51948
#NA's - station	16	12.68750	6.78939	6	32
Proportion NA - station	16	0.20464	0.10951	0.09677	0.51613

We can see that mean proportion of missing recordings by individual was 21.35 % (range 7.79 % to 51.95 %) for the dataset consisting of all measurements and the mean proportion of missing values was 20.46 % (range 9.67 % to 51.61 %) for the dataset consisting only of measurements from J. G. Mendel Czech Antarctic Station on Antarctica.

S5 Mean sleep heart rate trajectory

We distinquished two different time ranges to conduct our analysis. The first was the whole expedition between 20.12.2021 and 06.03.2022, the second was only the time spent at J. G. Mendel Czech Antarctic Station – from 01.01.2022 to 03.03.2022.

S5.1 All expedition

S5.1.1 Linear trend

The final model assumes heteroskedasticity and auto-correlation structure of the form AR(2). Below is shown only a part of the output from `summary()` of this model.

```
model <- gls(mean_HR ~ time + ID, data = data, na.action = na.omit,
          correlation = corARMA(p=2, form = ~time|ID),
          weights = varIdent(form = ~1|ID))
```

Table S5.1: Summary table of the final model. The values of regression coefficients, their standard errors and p-values are reported. In the last column adjusted p-values using Benjamini-Hochberg (FDR) correction are shown. We set subject ANT048 as a reference threshold for the intercept in the model.

	Value	Std.Error	p-value	p-adjusted
(Intercept)	56.069030	0.965894	<10^{-9}	<10^{-9}
time	−0.048542	0.010047	0.000001579	0.000003947
IDANT074	21.825880	1.424995	<10^{-9}	<10^{-9}
IDANT106	6.194070	1.470239	0.000027598	0.000053074
IDANT129	−0.718700	1.360444	0.597425688	0.878567188
IDANT143	19.442300	1.312479	<10^{-9}	<10^{-9}
IDANT145	5.394981	1.243888	0.000015970	0.000033271
IDANT147	5.251706	1.293795	0.000053295	0.000095169
IDANT148	15.345370	1.502256	<10^{-9}	<10^{-9}
IDANT153	14.003950	1.248781	<10^{-9}	<10^{-9}
IDANT159	8.228754	1.268320	<10^{-9}	<10^{-9}
IDANT160	15.227500	1.459244	<10^{-9}	<10^{-9}
IDANT165	4.648944	1.524444	0.002354860	0.003924767
IDANT176	−1.727768	1.102437	0.117394054	0.183428209
IDANT209	−5.258087	1.102124	0.000002122	0.000004822
IDANT211	8.617571	1.155540	<10^{-9}	<10^{-9}
IDANT217	7.106839	1.368658	0.000000254	0.000000705

Now we perform *model diagnostics*.

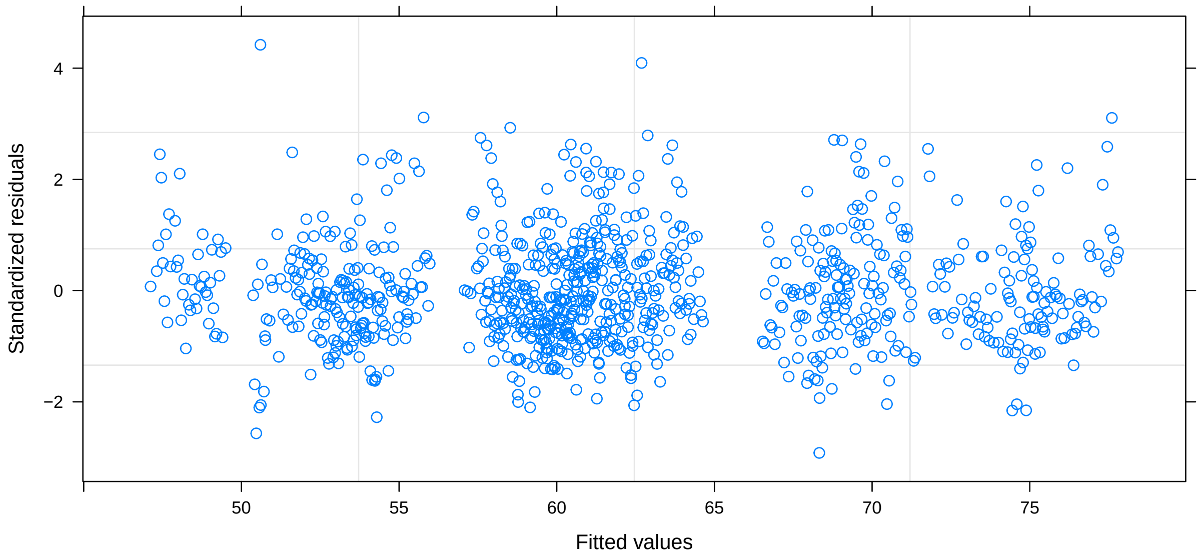

Figure S5.1: Fitted values against standardized residuals. The residuals appear to be randomly distributed around the fitted values.

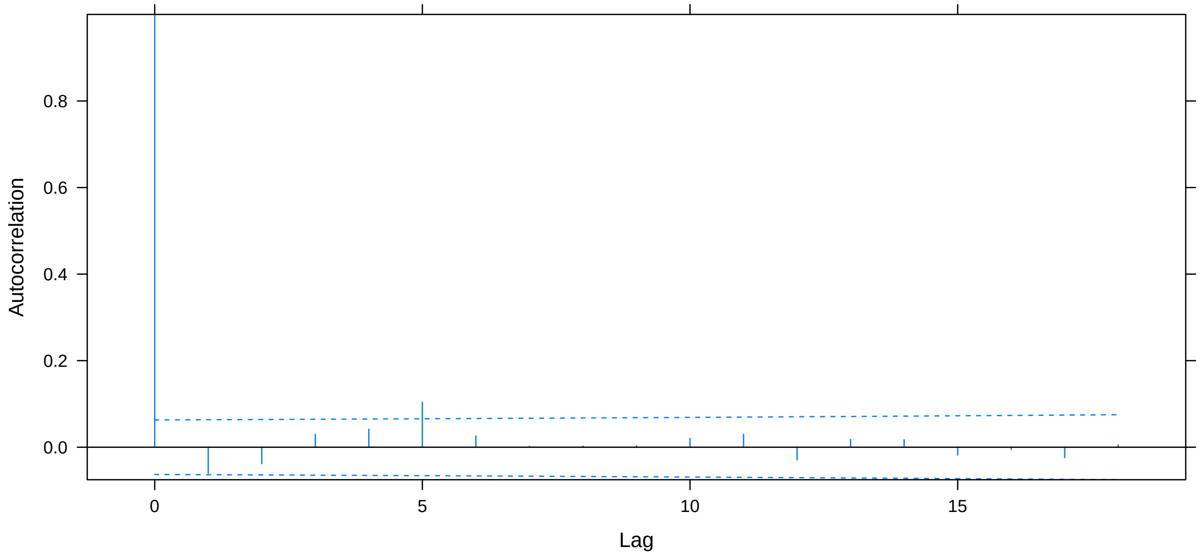

Figure S5.2: Graph of autocorrelation function (ACF) of model residuals with 95% confidence intervals. The residuals appear to be independent.

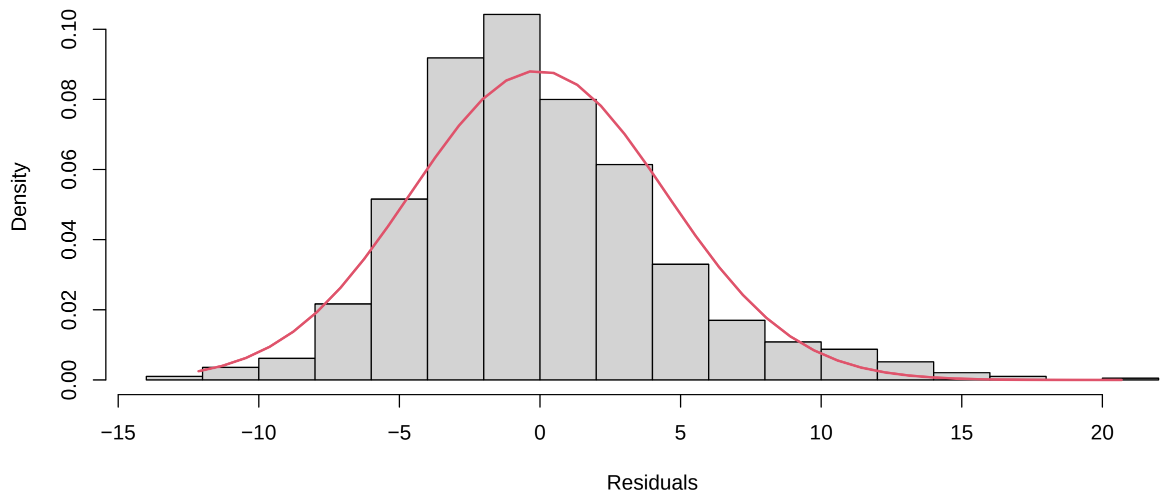

Figure S5.3: Histogram of residuals of the final model. The red solid line shows the density function of normal distribution with parameters calculated from the residuals. The residuals can be seen as normally distributed.

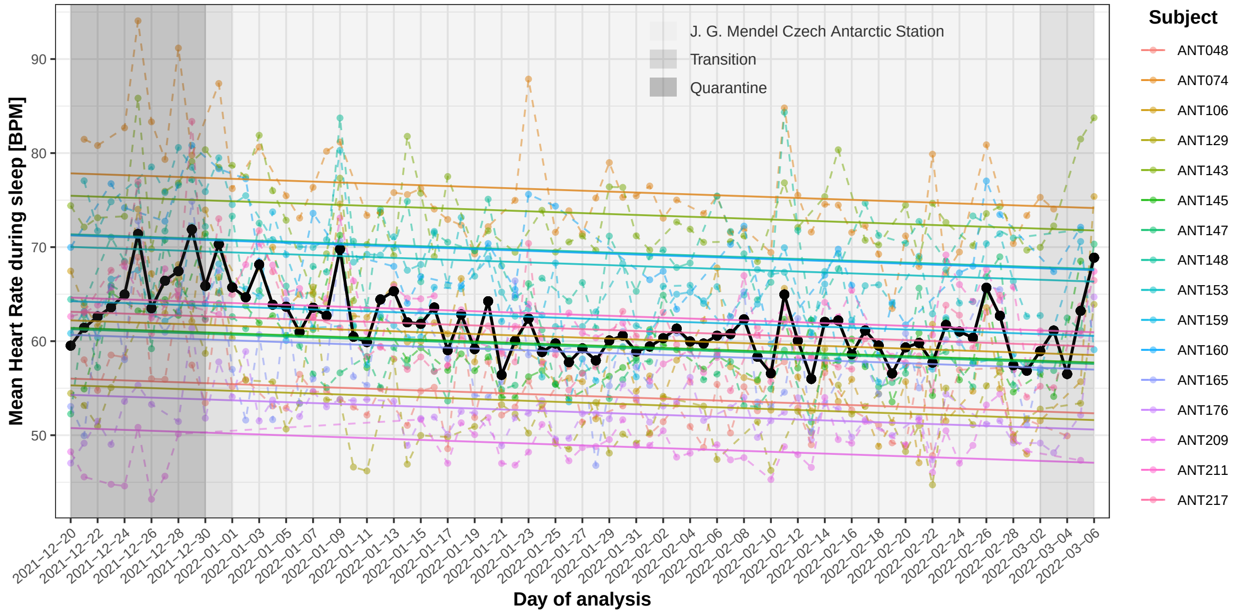

Figure S5.4: Individual trajectories of mean sleep heart rate during quarantine, transition to J. G. Mendel Czech Antarctic Station, stay at J. G. Mendel Czech Antarctic Station, and transition from J. G. Mendel Czech Antarctic Station Black depicts mean values across all participants.

S5.1.2 Quadratic trend

From the figure of individual trajectories with linear trend above we can see that at the end of the expedition the mean heart rate during sleep has tendency to increase. Therefore, we also fit the model as-

suming quadratic dependence of mean heart rate during sleep on time. In this model we again assume heteroskedasticity and auto-correlation structure of form AR(2).

```
model.2 <- gls(mean_HR ~ time + I(time^2) + ID, data = data, na.action = na.omit,
               correlation = corARMA(p=2, form = ~time|ID),
               weights = varIdent(form = ~1|ID))
```

Table S5.2: Summary table of the final quadratic model. The values of regression coefficients, their standard errors corresponding and p-values are reported. In the last column adjusted p-values using Benjamini-Hochberg (FDR) correction are shown.

	Value	Std.Error	p-value	p-adjusted
(Intercept)	58.516390	0.966756	<10ˆ{-9}	<10ˆ{-9}
time	−0.225135	0.035560	<10ˆ{-9}	0.000000001
I(timeˆ2)	0.002230	0.000446	0.000000693	0.000001443
IDANT074	21.798510	1.253402	<10ˆ{-9}	<10ˆ{-9}
IDANT106	6.038224	1.303847	0.000004143	0.000007398
IDANT129	−0.862915	1.193788	0.469957488	0.652718734
IDANT143	19.285280	1.152734	<10ˆ{-9}	<10ˆ{-9}
IDANT145	5.345952	1.094838	0.000001226	0.000002358
IDANT147	5.127333	1.133159	0.000006809	0.000011349
IDANT148	15.310250	1.340540	<10ˆ{-9}	<10ˆ{-9}
IDANT153	14.027340	1.096513	<10ˆ{-9}	<10ˆ{-9}
IDANT159	8.111426	1.142747	<10ˆ{-9}	<10ˆ{-9}
IDANT160	15.076780	1.301814	<10ˆ{-9}	<10ˆ{-9}
IDANT165	4.632176	1.354354	0.000652334	0.001019272
IDANT176	−1.845643	0.987272	0.061869049	0.090983896
IDANT209	−5.303283	1.014315	0.000000210	0.000000478
IDANT211	8.507520	1.015958	<10ˆ{-9}	<10ˆ{-9}
IDANT217	7.012341	1.200392	0.000000007	0.000000018

Now we perform *model diagnostics* of the quadratic model.

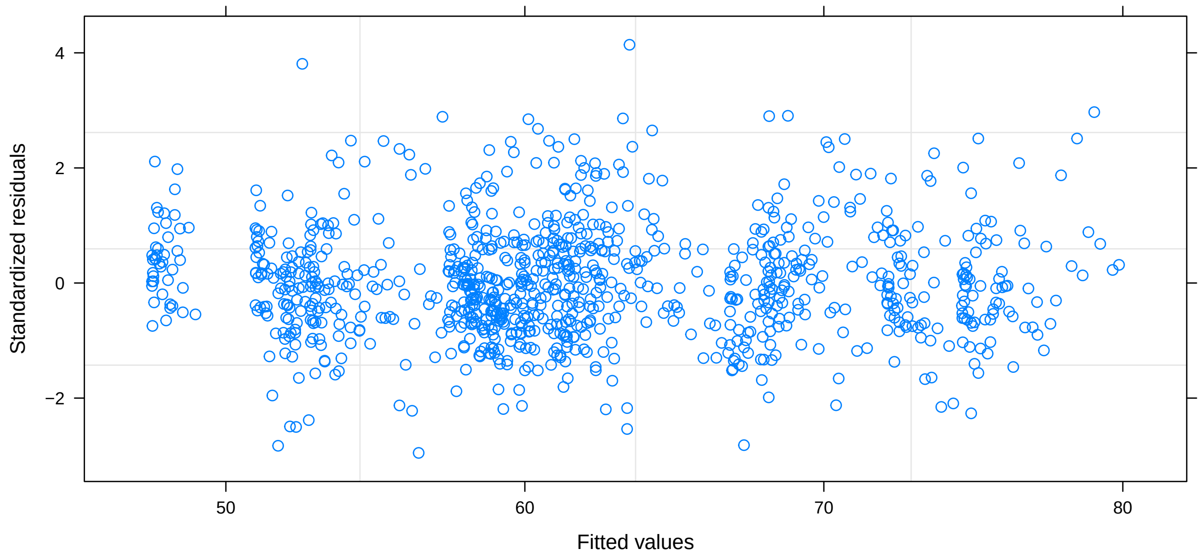

Figure S5.5: Fitted values against standardized residuals. The residuals appear to be randomly distributed around the fitted values.

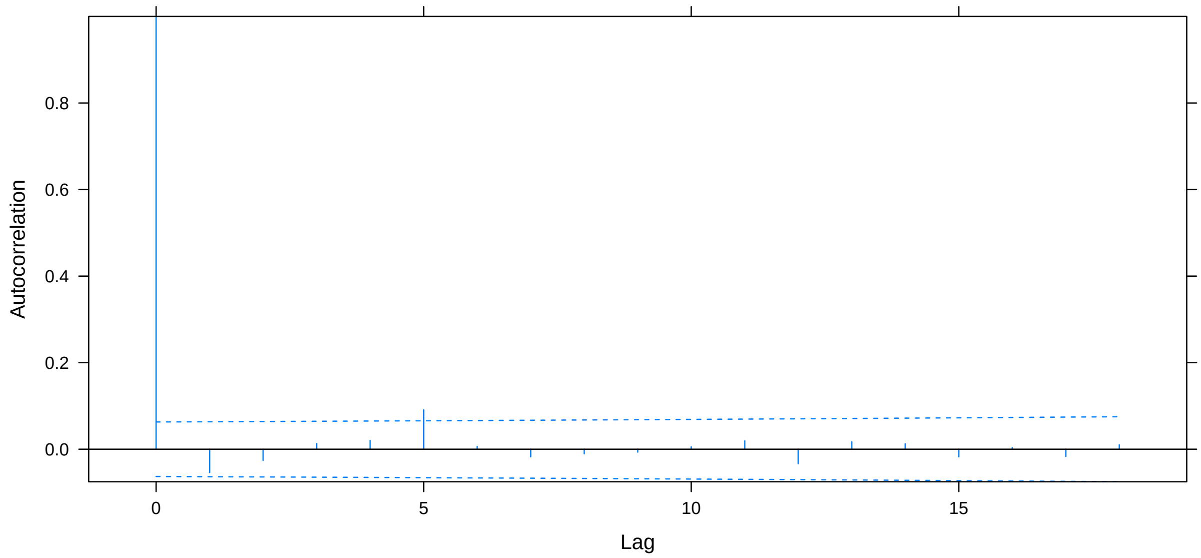

Figure S5.6: Graph of autocorrelation function (ACF) of model residuals with 95% confidence intervals. The residuals appear to be independent.

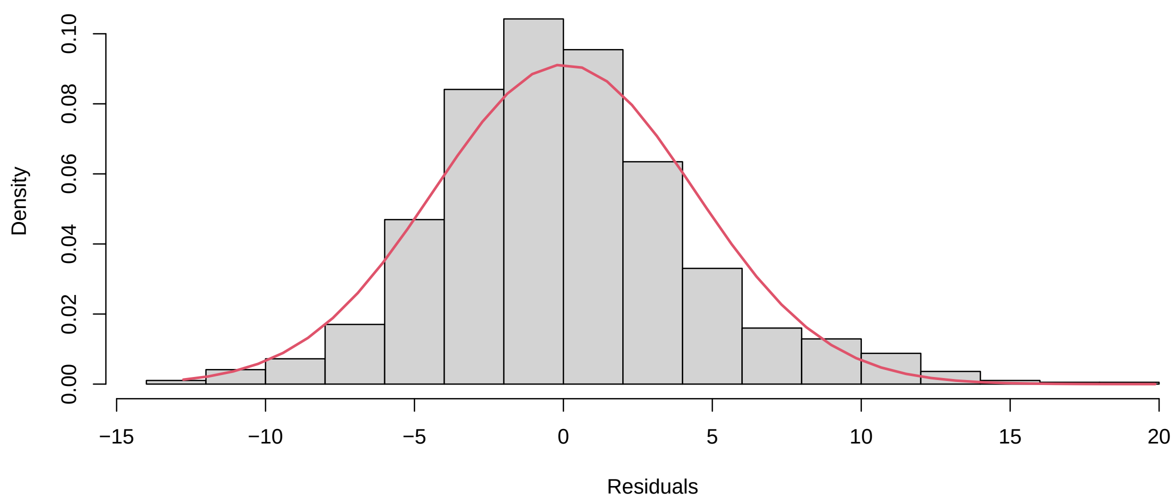

Figure S5.7: Histogram of residuals of the final model. The red solid line shows the density function of normal distribution with parameters calculated from the residuals. The residuals can be seen as normally distributed.

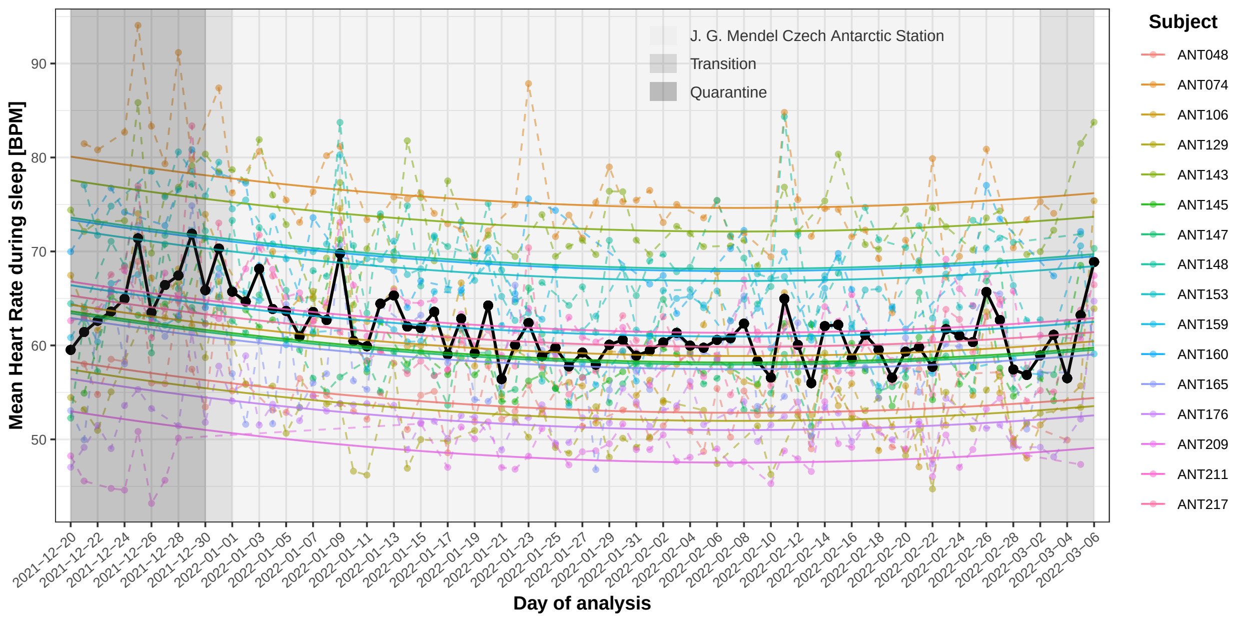

Figure S5.8: Individual trajectories of mean sleep heart rate during quarantine, transition to J. G. Mendel Czech Antarctic Station, stay at J. G. Mendel Czech Antarctic Station, and transition from J. G. Mendel Czech Antarctic Station assuming quadratic dependence of mean sleep heart rate on time. Black solid line depicts mean values across all participants.

S5.2 Station only

```
model.A <- gls(mean_HR ~ time + ID, data = data, na.action = na.omit,
               correlation = corARMA(p=2, form = ~time|ID),
               weights = varIdent(form = ~1|ID))
```

Table S5.3: Summary table of the final linear model only for time spent on J. G. Mendel Czech Antarctic Station in Antarctica. The values of regression coefficients, their standard errors and corresponding p-values are reported. In the last column adjusted p-values using Benjamini-Hochberg (FDR) correction are shown.

	Value	Std.Error	p-value	p-adjusted
(Intercept)	55.161460	0.663059	<10^{-9}	<10^{-9}
time	−0.056984	0.009211	<10^{-9}	0.000000002
IDANT074	21.062690	0.964555	<10^{-9}	<10^{-9}
IDANT106	5.167449	1.073520	0.000001784	0.000003186
IDANT129	−1.305009	0.959816	0.174339106	0.256381038
IDANT143	19.209340	0.934606	<10^{-9}	<10^{-9}
IDANT145	5.241952	0.851766	0.000000001	0.000000003
IDANT147	4.951833	0.908084	0.000000067	0.000000139
IDANT148	16.044280	1.163814	<10^{-9}	<10^{-9}
IDANT153	13.391190	0.904045	<10^{-9}	<10^{-9}
IDANT159	8.506638	0.969116	<10^{-9}	<10^{-9}
IDANT160	14.679020	1.199221	<10^{-9}	<10^{-9}
IDANT165	4.871326	1.083994	0.000008065	0.000013442
IDANT176	−1.320880	0.706399	0.061879066	0.096686040
IDANT209	−3.757825	0.772270	0.000001381	0.000002656
IDANT211	9.033258	0.876934	<10^{-9}	<10^{-9}
IDANT217	6.360617	0.866512	<10^{-9}	<10^{-9}

Now we perform *model diagnostics* of the linear model assuming independence between subjects, heteroskedasticity, normality of residuals.

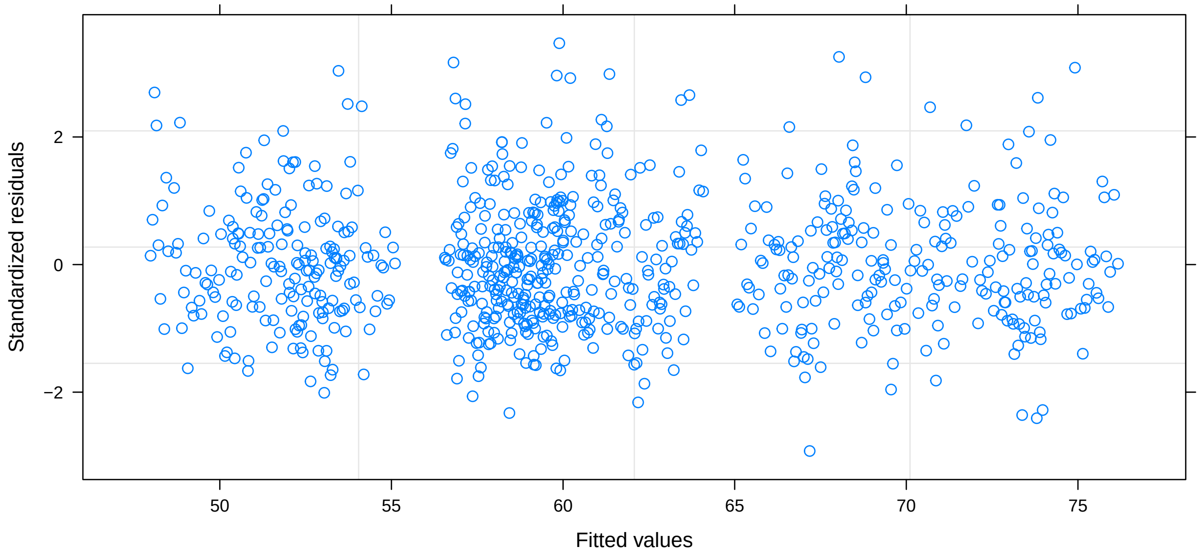

Figure S5.9: Fitted values against standardized residuals. The residuals appear to be randomly distributed around the fitted values.

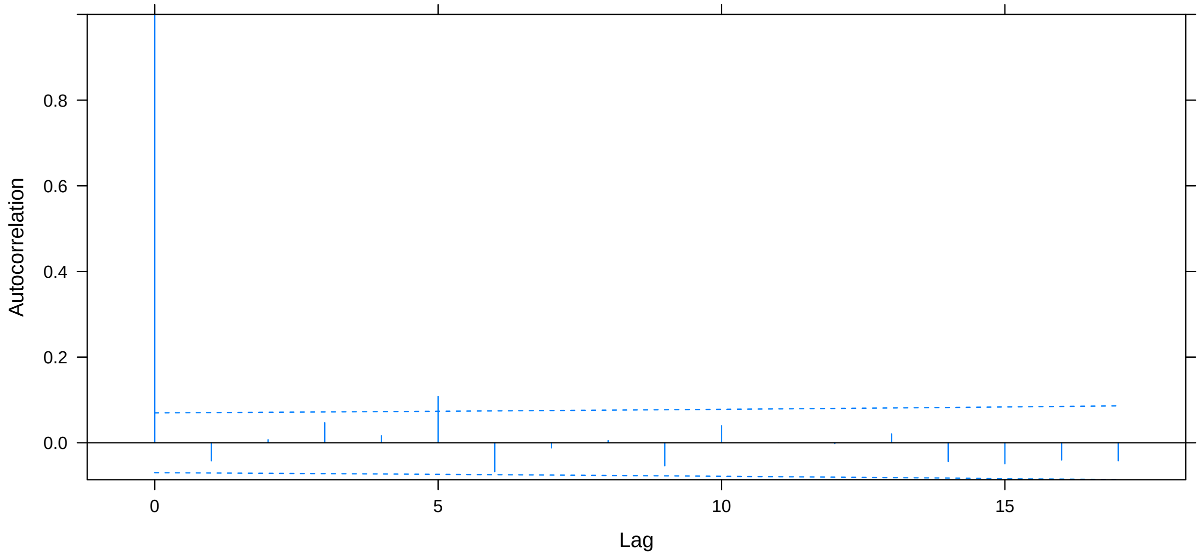

Figure S5.10: Graph of autocorrelation function (ACF) of model residuals with 95% confidence intervals. The residuals appear to be independent.

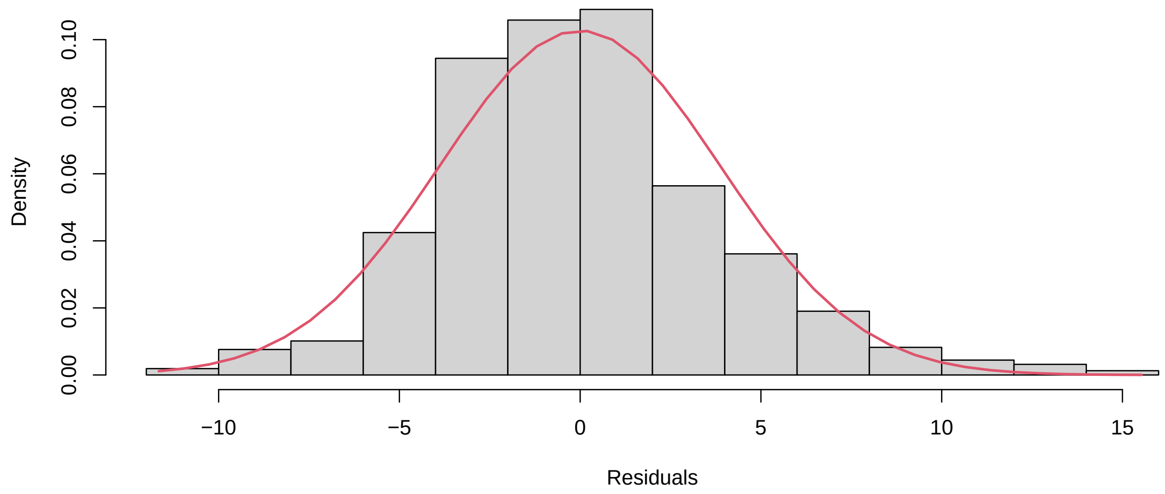

Figure S5.11: Histogram of residuals of the final model. The red solid line shows the density function of normal distribution with parameters calculated from the residuals. The residuals can be seen as normally distributed.

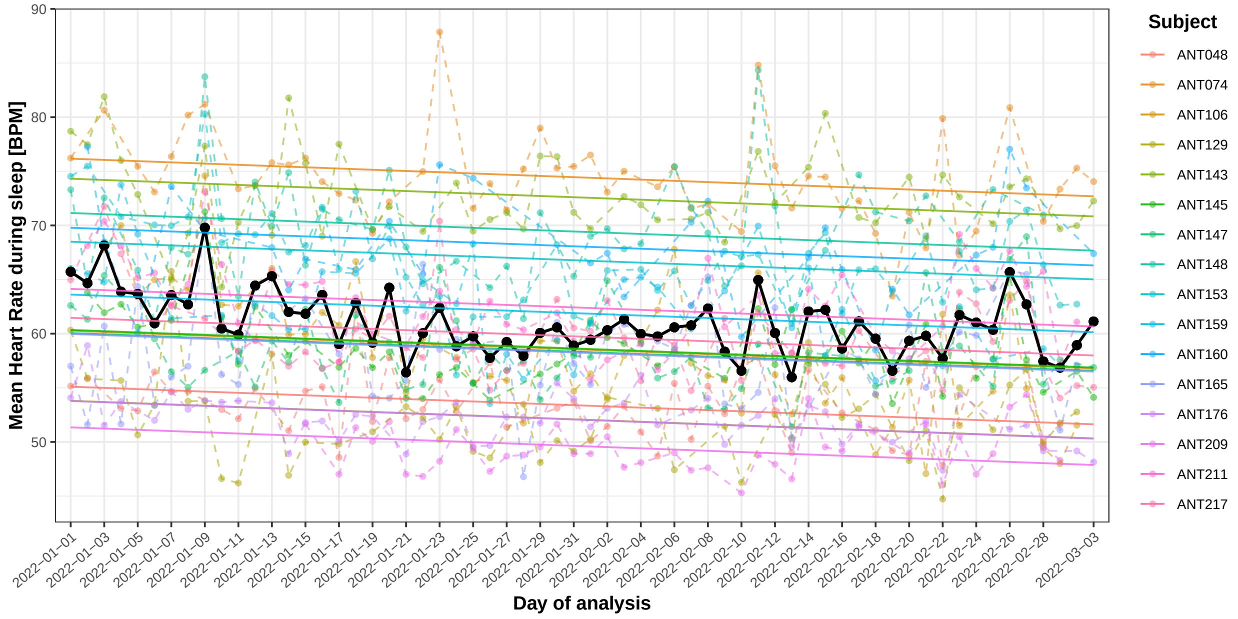

Figure S5.12: Individual trajectories of mean sleep heart rate during the stay at J. G. Mendel Czech Antarctic Station assuming linear dependence of mean sleep heart rate on time. Black solid line depicts mean values across all participants.

S6 Mean sleep heart rate and co-factors

S6.1 All expedition

```
model.complex <- gls(mean_HR ~ time + duration + Age + Sex + First.timer + BMI,
                     data = data.full, na.action = na.omit,
                     correlation = corARMA(p=2, form = ~time|ID),
                     weights = varIdent(form = ~1|ID))
```

Table S6.1: Summary table of the final complex model with co-factors. The values of regression coefficients, their standard errors and corresponding p-values are reported. In the last column adjusted p-values using Benjamini-Hochberg (FDR) correction are shown.

	Value	Std.Error	p-value	p-adjusted
(Intercept)	25.712120	10.223000	0.012072038	0.075450235
time	−0.012730	0.027986	0.649326745	1.000000000
duration	−0.478009	0.081861	0.000000007	0.000000091
Age	0.260097	0.140347	0.064175335	0.320876676
SexMale	2.470907	2.156141	0.252105357	1.000000000
First.timerYes	16.416120	2.765506	0.000000004	0.000000091
BMI	0.860900	0.298205	0.003983076	0.033192297

Now we perform *model diagnostics* of the complex model of mean sleep heart rate with co-factors assuming independence between subjects, heteroskedasticity and normality of residuals.

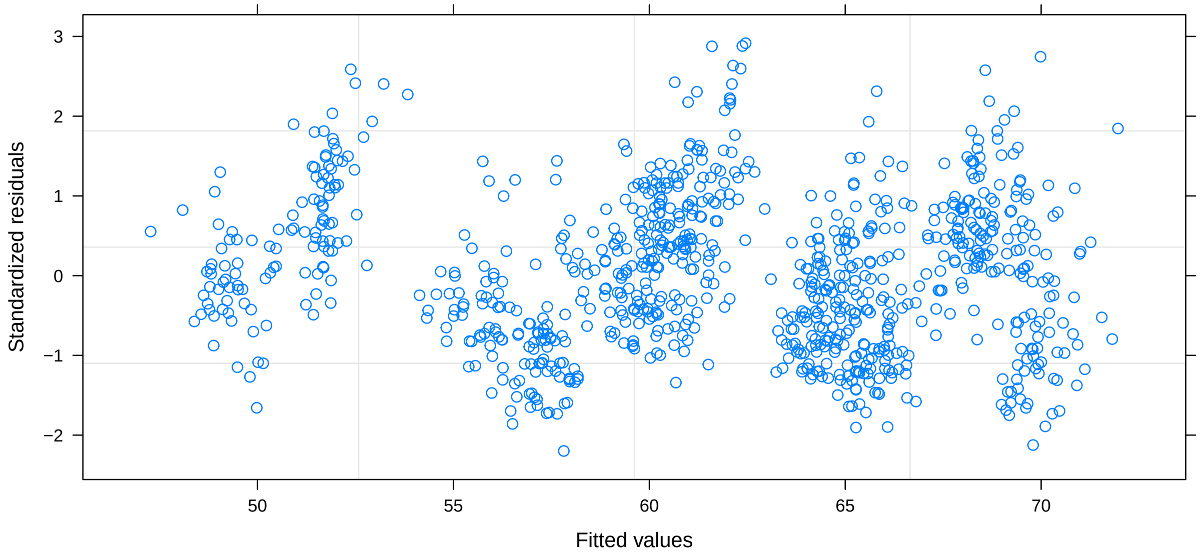

Figure S6.1: Fitted values against standardized residuals. The residuals appear to be randomly distributed around the fitted values.

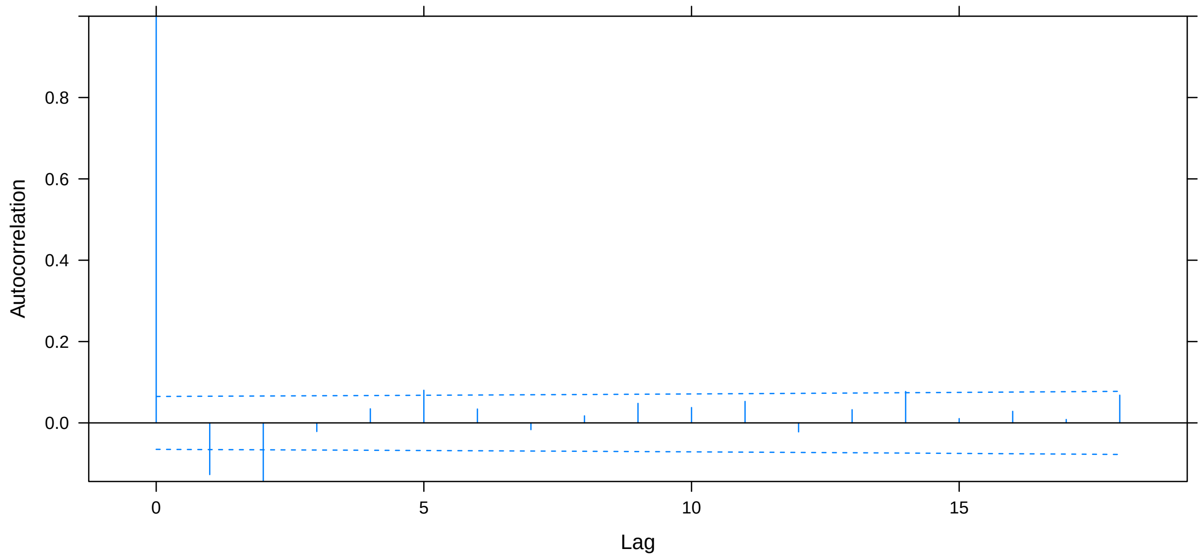

Figure S6.2: Graph of autocorrelation function (ACF) of model residuals with 95% confidence intervals. The residuals appear to be independent.

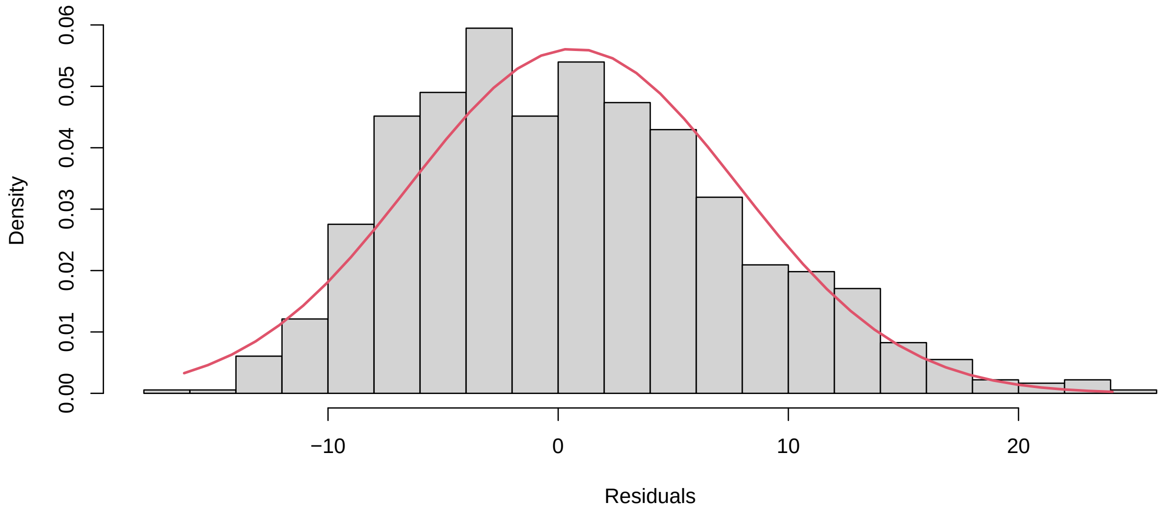

Figure S6.3: Histogram of residuals of the final complex model with co-factors. The red solid line shows the density function of normal distribution with parameters calculated from the residuals. The residuals can be seen as normally distributed.

S6.2 Station only

```
model.complex.2 <- gls(mean_HR ~ time + duration + Age + Sex + First.timer + BMI,
                       data = data.full, na.action = na.omit,
                       correlation = corARMA(p=2, form = ~time|ID),
                       weights = varIdent(form = ~1|ID))
```

Table S6.2: Summary table of the final complex model with co-factors for J. G. Mendel Czech Antarctic Station only. The values of regression coefficients, their standard errors and corresponding p-values are reported. In the last column adjusted p-values using Benjamini-Hochberg (FDR) correction are shown.

	Value	Std.Error	p-value	p-adjusted
(Intercept)	28.535130	9.539497	0.002871432	0.017946452
time	−0.087240	0.031972	0.006512619	0.032563096
duration	−0.394754	0.081638	0.000001620	0.000020246
Age	0.245574	0.126611	0.052812225	0.220050937
SexMale	0.704202	1.957539	0.719146362	1.000000000
First.timerYes	15.739000	2.609154	0.000000003	0.000000064
BMI	0.850139	0.269961	0.001704075	0.014200621

Now we perform *model diagnostics* of the complex model of mean sleep heart rate with co-factors assuming independence between subjects, heteroskedasticity and normality of residuals.

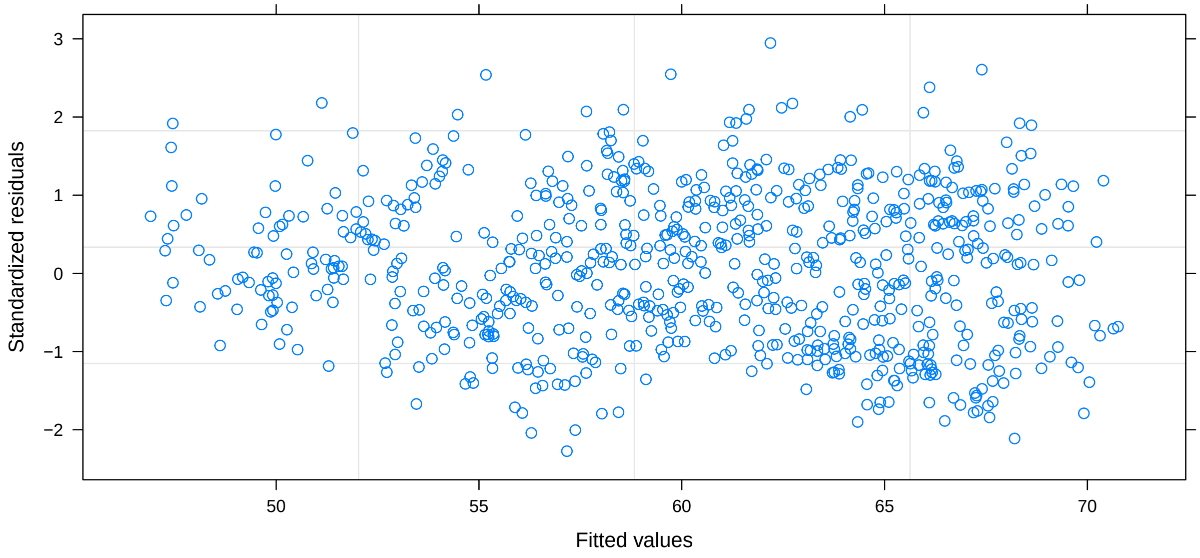

Figure S6.4: Fitted values against standardized residuals. The residuals appear to be randomly distributed around the fitted values.

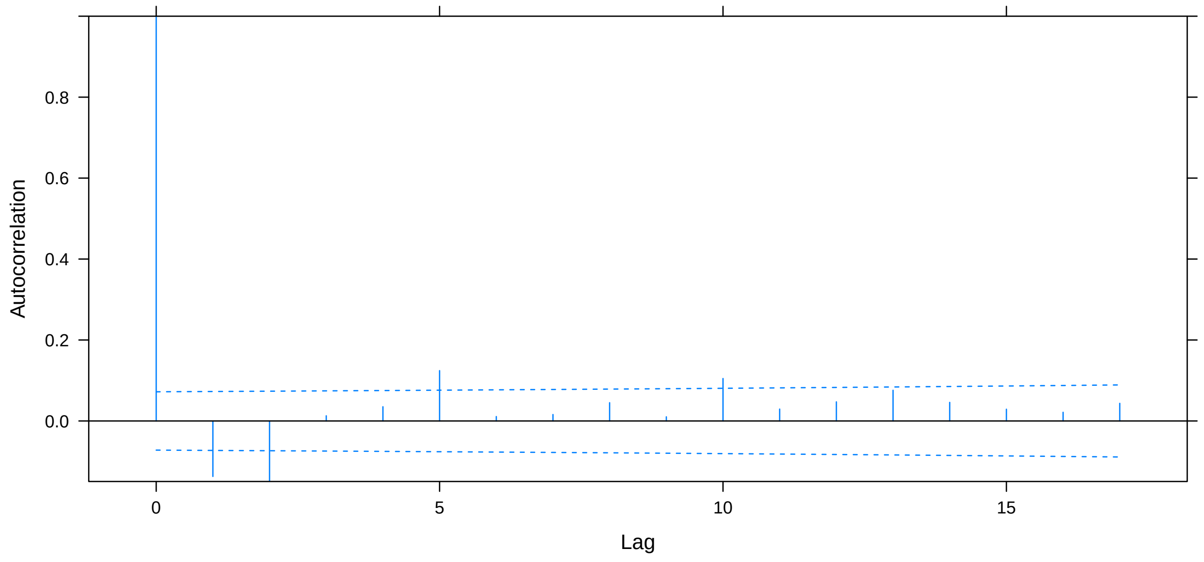

Figure S6.5: Graph of autocorrelation function (ACF) of model residuals with 95% confidence intervals. The residuals appear to be independent.

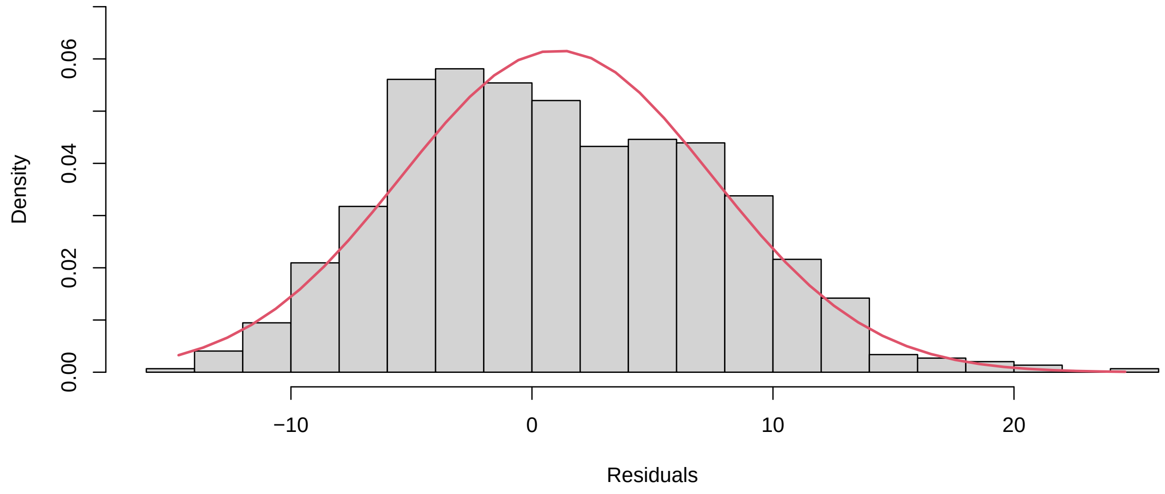

Figure S6.6: Histogram of residuals of the final complex model with co-factors. The red solid line shows the density function of normal distribution with parameters calculated from the residuals. The residuals appear to be normally distributed.

S7 Mood trajectory

S7.1 Tension

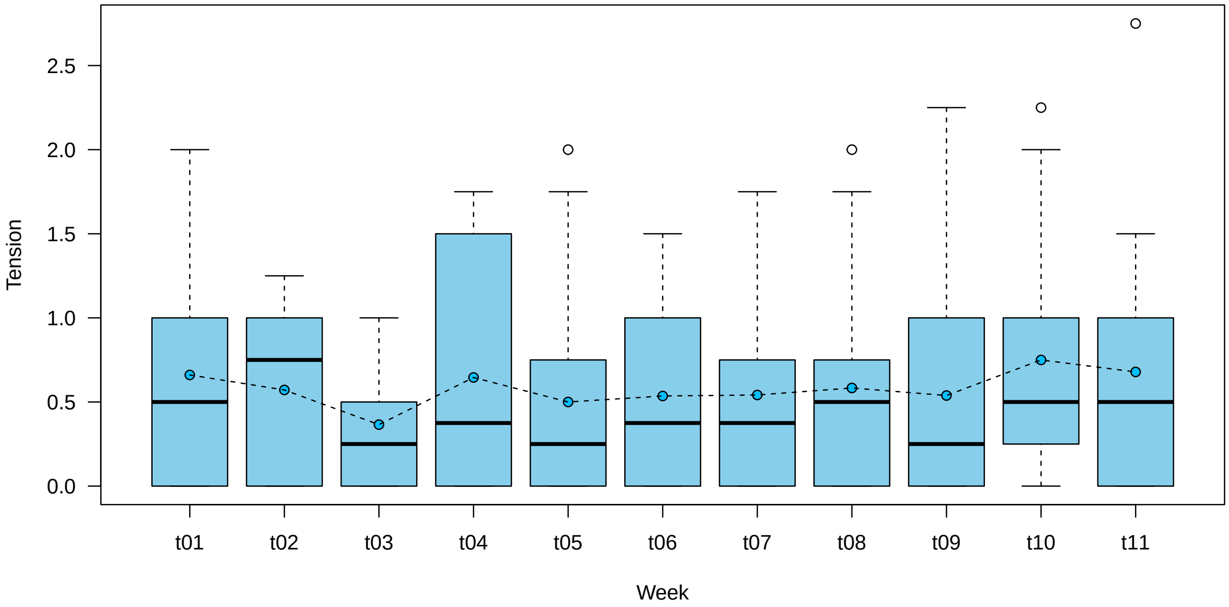

Figure S7.1: Box plots for week-by-week score of POMS questionnaire (Tension). The mean values are represented by a blue point, median values are represented by a dash.

For modeling the dependence of Tension on time, sex, age, BMI and previous experience with expedition (variable First.timer) we assumed intra-subject correlation of the form AR(1). We compared two models, one assuming homoskedasticity and the other heteroskedasticity, corresponding p-value of the comparison of these two models (using `anova()` function in `R`) is 0.0061476, therefore we continued with model assuming heteroskedasticity.

Table S7.1: Summary table of the model of dependence of Tension on time, sex, BMI, age and previous experience with expedition (factor First.timer). The values of regression coefficients, their standard errors and corresponding p-values are reported. In the last column adjusted p-values using Benjamini-Hochberg (FDR) correction are shown.

	Value	Std.Error	p-value	p-adjusted
(Intercept)	−1.648578	0.743335	0.028345531	0.236212757
time	0.022125	0.020598	0.284803886	1.000000000
SexMale	−0.738623	0.141115	0.000000666	0.000016642
Age	−0.000717	0.010536	0.945850460	1.000000000
First.timerYes	0.322192	0.221295	0.147877100	0.924231874
BMI	0.094049	0.024324	0.000175305	0.002191310

S7.2 Depression

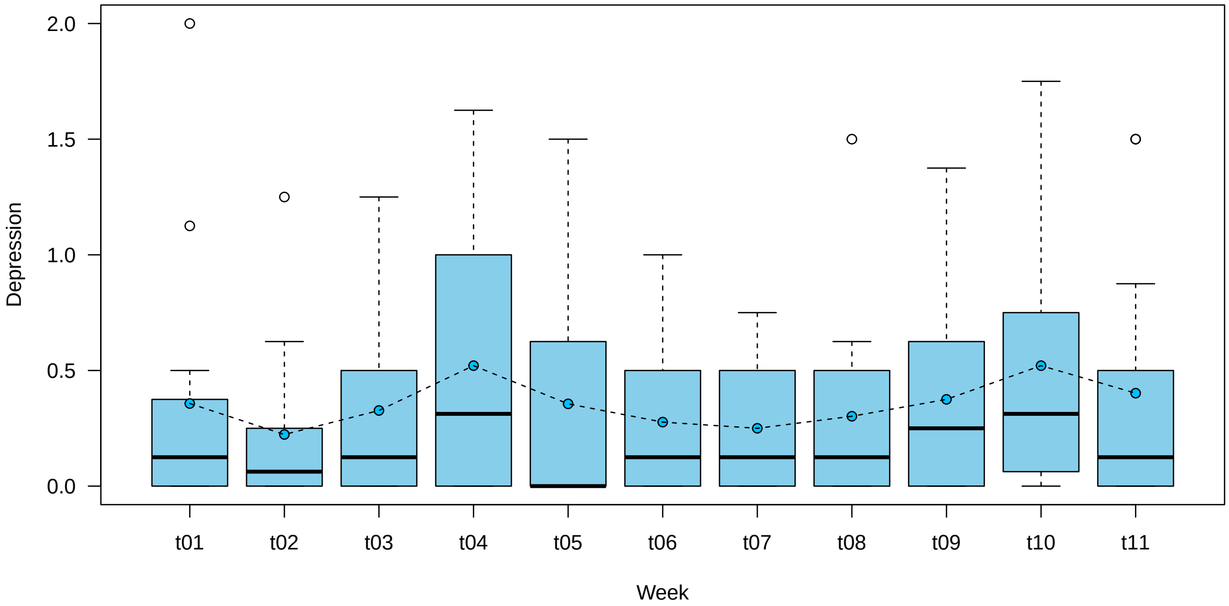

Figure S7.2: Box plots for week-by-week score of POMS questionnaire (Depression). The mean values are represented by a blue point, median values are represented by a dash.

For modeling the dependence of Depression on time, sex, age, BMI and previous experience with expedition (variable First.timer) we assumed intra-subject correlation of the form AR(1). We compared two models, one assuming homoskedasticity and the other heteroskedasticity, corresponding p-value of the comparison of these two models (using `anova()` function in `R`) is 0.0047553, therefore we continued with model assuming heteroskedasticity.

Table S7.2: Summary table of the model of dependence of Depression on time, sex, BMI, age and previous experience with expedition (factor First.timer). The values of regression coefficients, their standard errors and corresponding p-values are reported. In the last column adjusted p-values using Benjamini-Hochberg (FDR) correction are shown.

	Value	Std.Error	p-value	p-adjusted
(Intercept)	−1.635660	0.629879	0.010529024	0.087741864
time	0.010563	0.013010	0.418385699	1.000000000
SexMale	−0.524206	0.120859	0.000029261	0.000731515
Age	0.008479	0.008269	0.307184106	1.000000000
First.timerYes	0.404414	0.179663	0.026120894	0.163255590
BMI	0.069958	0.018610	0.000259658	0.003245720

S7.3 Anger

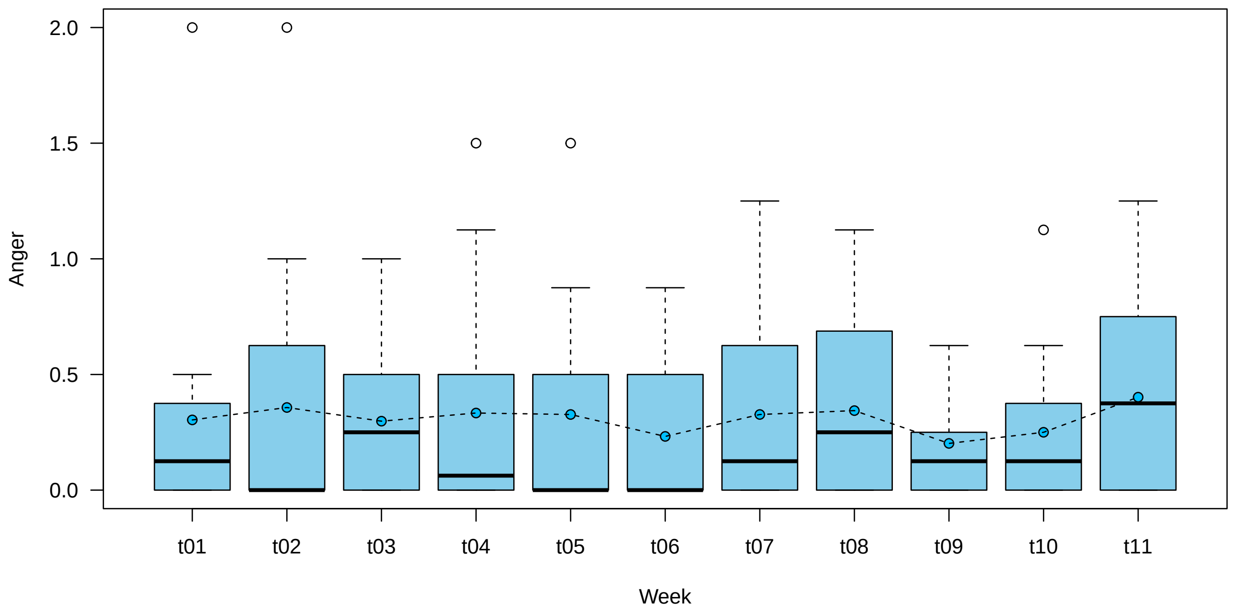

Figure S7.3: Box plots for week-by-week score of POMS questionnaire (Anger). The mean values are represented by a blue point, median values are represented by a dash.

For modeling the dependence of Anger on time, sex, age, BMI and previous experience with expedition (variable First.timer) we assumed intra-subject correlation of the form AR(1). We compared two models, one assuming homoskedasticity and the other heteroskedasticity, corresponding p-value of the comparison of these two models (using `anova()` function in `R`) is 1.0339943×10^{-4}, therefore we continued with model assuming heteroskedasticity.

Table S7.3: Summary table of the model of dependence of Anger on time, sex, BMI, age and previous experience with expedition (factor First.timer). The values of regression coefficients, their standard errors and corresponding p-values are reported. In the last column adjusted p-values using Benjamini-Hochberg (FDR) correction are shown.

	Value	Std.Error	p-value	p-adjusted
(Intercept)	−0.740905	0.443407	0.097177442	0.607359013
time	0.000509	0.009125	0.955634762	1.000000000
SexMale	−0.208694	0.085169	0.015623023	0.130191861
Age	−0.015465	0.006262	0.014840321	0.130191861
First.timerYes	−0.046389	0.131627	0.725095858	1.000000000
BMI	0.073655	0.014289	0.000000938	0.000023462

S7.4 Vigour

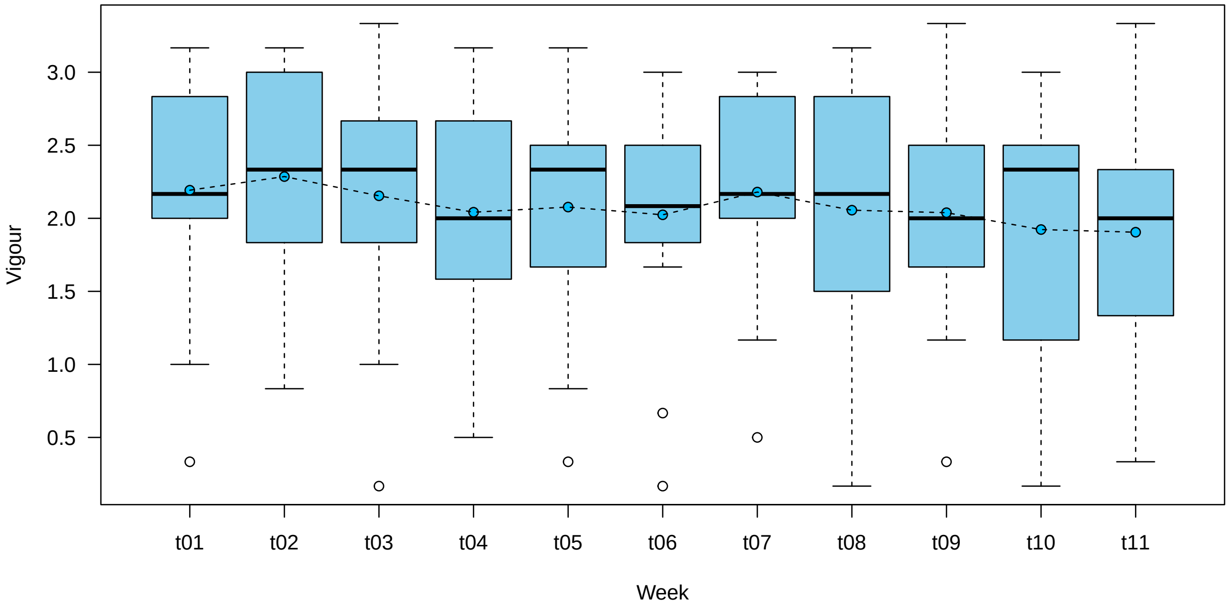

Figure S7.4: Box plots for week-by-week score of POMS questionnaire (Vigour). The mean values are represented by a blue point, median values are represented by a dash.

For modeling the dependence of Tension on time, sex, age, BMI and previous experience with expedition (variable First.timer) we assumed intra-subject correlation of the form AR(1). We compared two models, one assuming homoskedasticity and the other heteroskedasticity, corresponding p-value of the comparison of these two models (using `anova()` function in `R`) is 0.1026008, therefore we continued with the simple model assuming homoskedasticity.

Table S7.4: Summary table of the model of dependence of Vigour on time, sex, BMI, age and previous experience with expedition (factor First.timer). The values of regression coefficients, their standard errors and corresponding p-values are reported. In the last column adjusted p-values using Benjamini-Hochberg (FDR) correction are shown.

	Value	Std.Error	p-value	p-adjusted
(Intercept)	−1.618570	1.394299	0.247863698	1.000000000
time	−0.027012	0.021866	0.218964035	1.000000000
SexMale	−0.994588	0.267800	0.000303476	0.003793456
Age	0.083060	0.018147	0.000011014	0.000275351
First.timerYes	0.609804	0.390794	0.121128961	1.000000000
BMI	0.055125	0.041167	0.182926517	1.000000000

S7.5 Fatigue

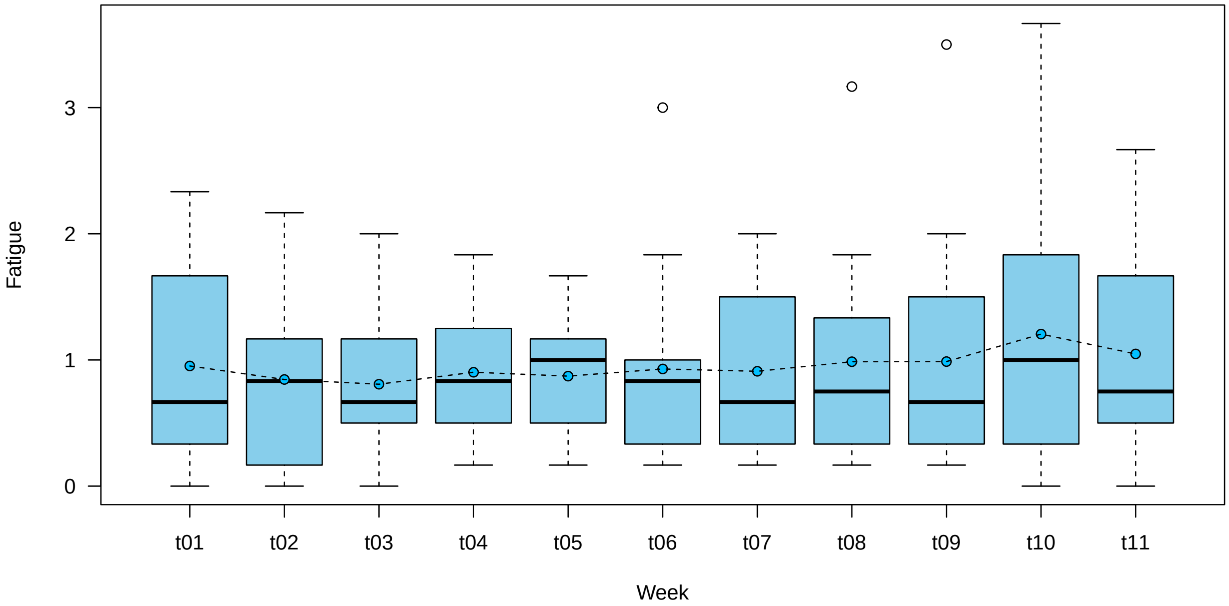

Figure S7.5: Box plots for week-by-week score of POMS questionnaire (Fatigue). The mean values are represented by a blue point, median values are represented by a dash.

For modeling the dependence of Fatigue on time, sex, age, BMI and previous experience with expedition (variable First.timer) we assumed intra-subject correlation of the form AR(1). We compared two models, one assuming homoskedasticity and the other heteroskedasticity, corresponding p-value of the comparison of these two models (using `anova()` function in `R`) is 0.0141204, therefore we continued with model assuming heteroskedasticity.

Table S7.5: Summary table of the model of dependence of Fatigue on time, sex, BMI, age and previous experience with expedition (factor First.timer). The values of regression coefficients, their standard errors and corresponding p-values are reported. In the last column adjusted p-values using Benjamini-Hochberg (FDR) correction are shown.

	Value	Std.Error	p-value	p-adjusted
(Intercept)	−0.968699	0.938751	0.304063694	1.000000000
time	0.018490	0.018893	0.329597261	1.000000000
SexMale	−0.383360	0.169127	0.025086657	0.209055474
Age	−0.027541	0.011800	0.021154625	0.209055474
First.timerYes	−0.152020	0.267100	0.570250952	1.000000000
BMI	0.128785	0.027242	0.000005905	0.000147621

S7.6 Confusion

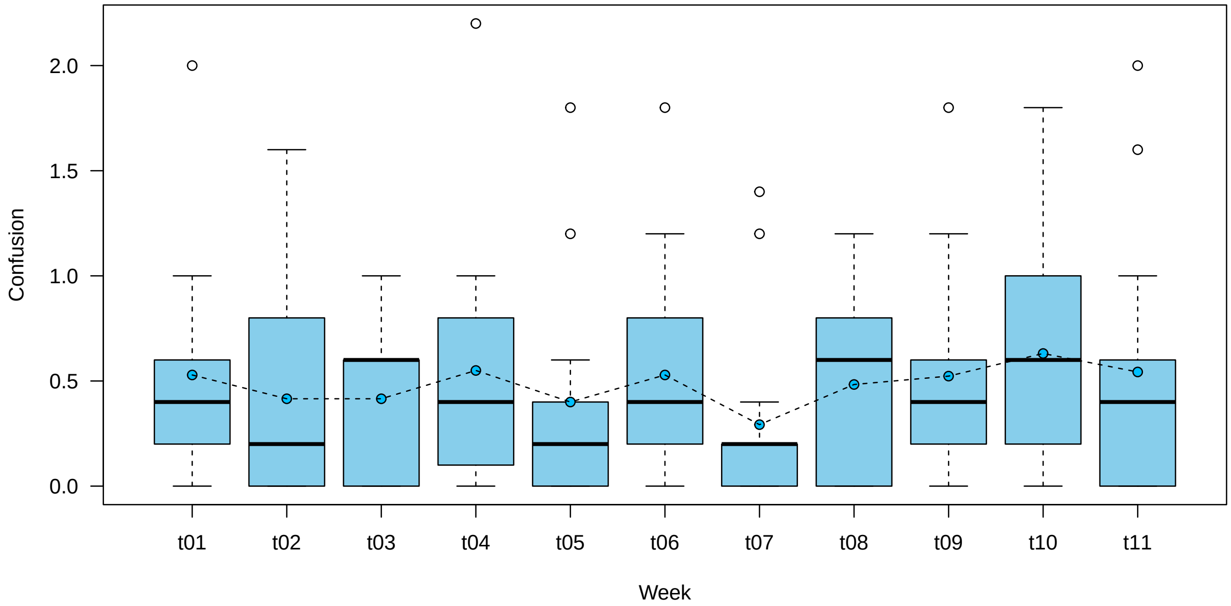

Figure S7.6: Box plots for week-by-week score of POMS questionnaire (Confusion). The mean values are represented by a blue point, median values are represented by a dash.

For modeling the dependence of Confusion on time, sex, age, BMI and previous experience with expedition (variable First.timer) we assumed intra-subject correlation of the form AR(1). We compared two models, one assuming homoskedasticity and the other heteroskedasticity, corresponding p-value of the comparison of these two models (using `anova()` function in `R`) is 0.4140313, therefore we continued with the simple model assuming homoskedasticity.

Table S7.6: Summary table of the model of dependence of Confusion on time, sex, BMI, age and previous experience with expedition (factor First.timer). The values of regression coefficients, their standard errors and corresponding p-values are reported. In the last column adjusted p-values using Benjamini-Hochberg (FDR) correction are shown.

	Value	Std.Error	p-value	p-adjusted
(Intercept)	−1.005578	1.002691	0.317826211	1.00000000
time	0.002489	0.016525	0.880517218	1.00000000
SexMale	−0.629742	0.192536	0.001380933	0.03452334
Age	−0.001103	0.013065	0.932836532	1.00000000
First.timerYes	0.292576	0.281617	0.300818966	1.00000000
BMI	0.074457	0.029608	0.013159650	0.16449562

S8 Mean sleep heart rate and mood

S8.1 All expedition

For modelling the dependence of mean sleep heart rate on all POMS subscales and other co-factors we used generalized least squares linear model (function `gls()`). We assumed different variance of residuals for each subject and also auto-correlation structure of form AR(1) with dependence on time.

```
model.full.1 <- gls(mean_HR ~ Tension + Depression + Anger + Vigour +
                      Fatigue + Confusion
                     + time + Sex + Age + First.timer + BMI + duration,
                     data = data.full, na.action = na.omit,
                     weights = varIdent(form = ~1|ID),
                     cor = corAR1(form = ~time|ID))
```

Table S8.1: Summary table of the final complex model with co-factors and POMS subscales. The values of regression coefficients, their standard errors and corresponding p-values are reported. In the last column adjusted p-values using Benjamini-Hochberg (FDR) correction are shown.

	Value	Std.Error	p-value	p-adjusted
(Intercept)	38.805030	27.057820	0.154219037	0.69166167
Tension	0.098199	0.663426	0.882585209	1.00000000
Depression	−0.434317	0.847754	0.609404996	1.00000000
Anger	0.719895	0.909974	0.430491280	1.00000000
Vigour	−2.073438	0.576197	0.000471869	0.01179673
Fatigue	0.700162	0.484939	0.151486536	0.69166167
Confusion	−0.261575	0.735198	0.722645582	1.00000000
time	−0.263804	0.181084	0.147873061	0.69166167
SexMale	1.844074	5.861285	0.753615825	1.00000000
Age	0.241348	0.393194	0.540539689	1.00000000
First.timerYes	17.914540	6.906351	0.010711711	0.13389639
BMI	0.547729	0.800836	0.495373583	1.00000000
duration	−0.355399	0.254959	0.165998801	0.69166167

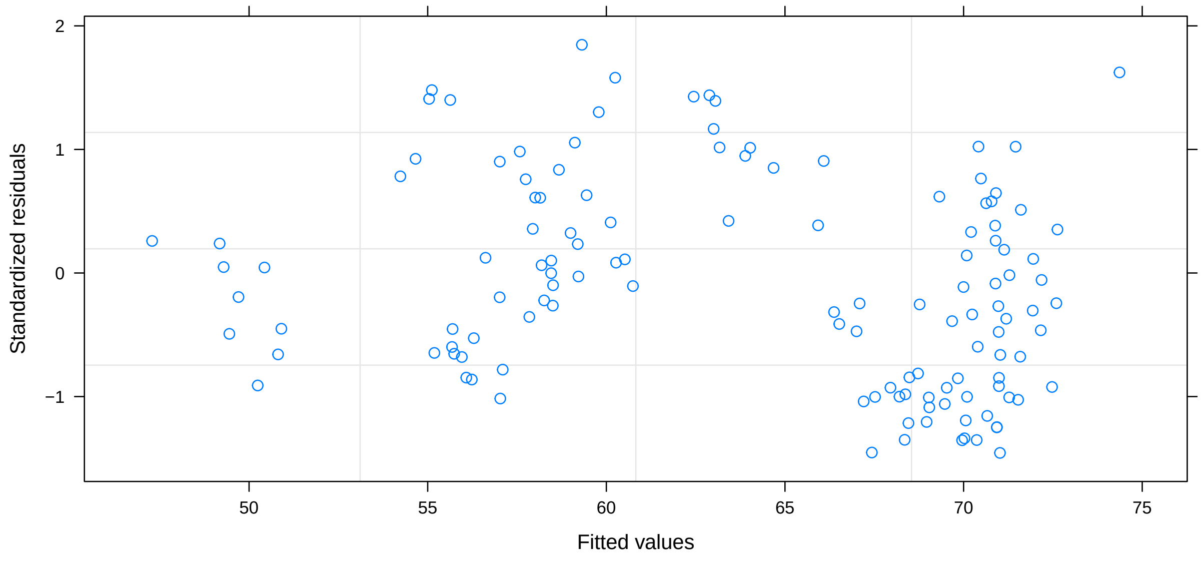

Figure S8.1: Standardized residuals against fitted values. The residuals appear to be randomly distributed around the fitted values.

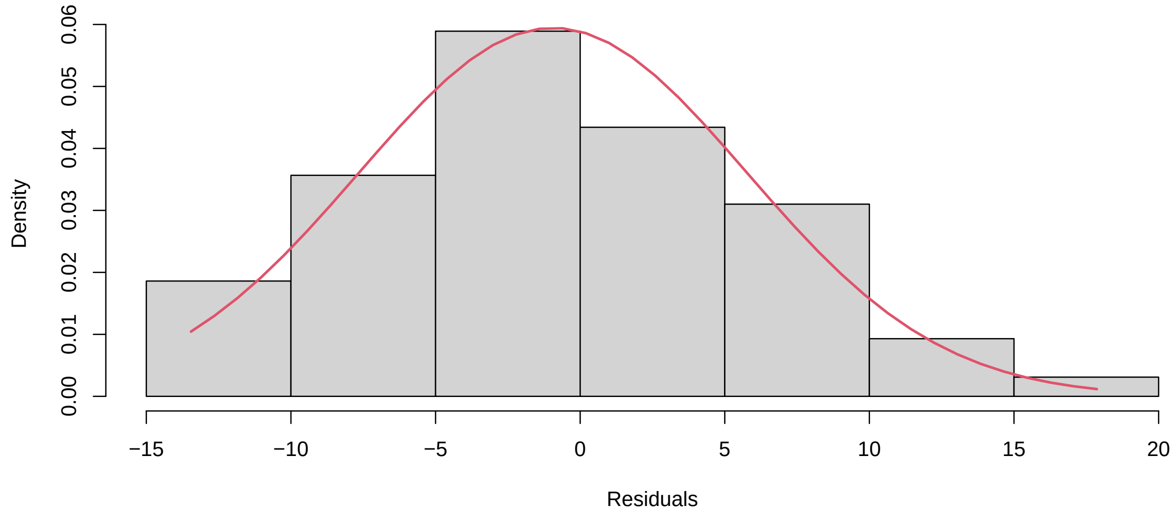

Figure S8.2: Histogram of residuals of the final complex model with co-factors and all POMS subscales. The red solid line shows the density function of normal distribution with parameters calculated from the residuals. The residuals appear to be normally distributed.

Normality of residuals appear to be normally distributed according to histogram above. The Shapiro-Wilk test of normality does not reject the null hypothesis, its p-value is 0.1901226.

S8.2 Station only

Now we consider only time spent in Antarctica (J. G. Mendel Czech Antarctic Station only). For modelling the dependence of mean sleep heart rate on all POMS subscales and other co-factors we again used generalized least squares linear model. We assumed different variance of residuals for each subject and also auto-correlation structure of form AR(1) with dependence on time.

```
model.full.2 <- gls(mean_HR ~ Tension + Depression + Anger + Vigour +
                      Fatigue + Confusion
                    + time + Sex + Age + First.timer + BMI + duration,
                    data = data.reduced.full, na.action = na.omit,
                    weights = varIdent(form = ~1|ID),
                    cor = corAR1(form = ~time|ID))
```

Table S8.2: Summary table of the final complex model with co-factors and POMS subscales for J. G. Mendel Czech Antarctic Station only. The values of regression coefficients, their standard errors and corresponding p-values are reported. In the last column adjusted p-values using Benjamini-Hochberg (FDR) correction are shown.

	Value	Std.Error	p-value	p-adjusted
(Intercept)	23.869980	25.725540	0.35633685	1.00000000
Tension	−0.138786	0.787788	0.86061561	1.00000000
Depression	−0.101760	0.848789	0.90487954	1.00000000
Anger	1.277376	0.955126	0.18498242	0.90202331
Vigour	−1.880903	0.766585	0.01637299	0.20466239
Fatigue	0.473003	0.541329	0.38492050	1.00000000
Confusion	−0.585421	0.755960	0.44103112	1.00000000
time	−0.338359	0.213690	0.11737378	0.90202331
SexMale	−2.274903	5.469674	0.67861644	1.00000000
Age	0.409866	0.328939	0.21648560	0.90202331
First.timerYes	21.237640	6.435317	0.00145841	0.03646025
BMI	0.916228	0.708237	0.19959549	0.90202331
duration	−0.134878	0.259828	0.60515630	1.00000000

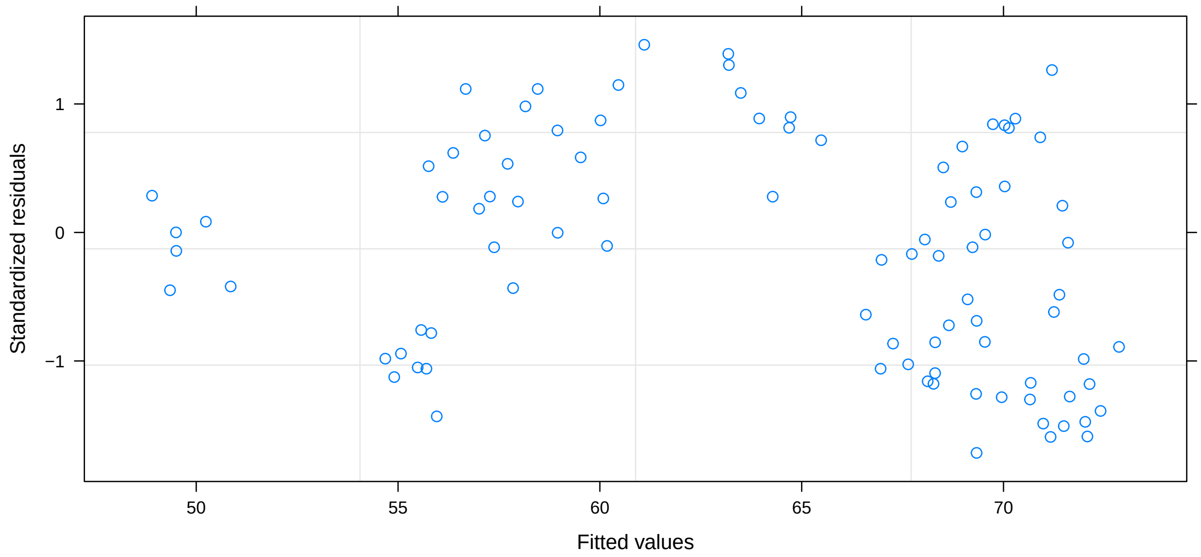

Figure S8.3: Plot of standardized residuals against fitted values. The residuals appear to be randomly distributed around the fitted values.

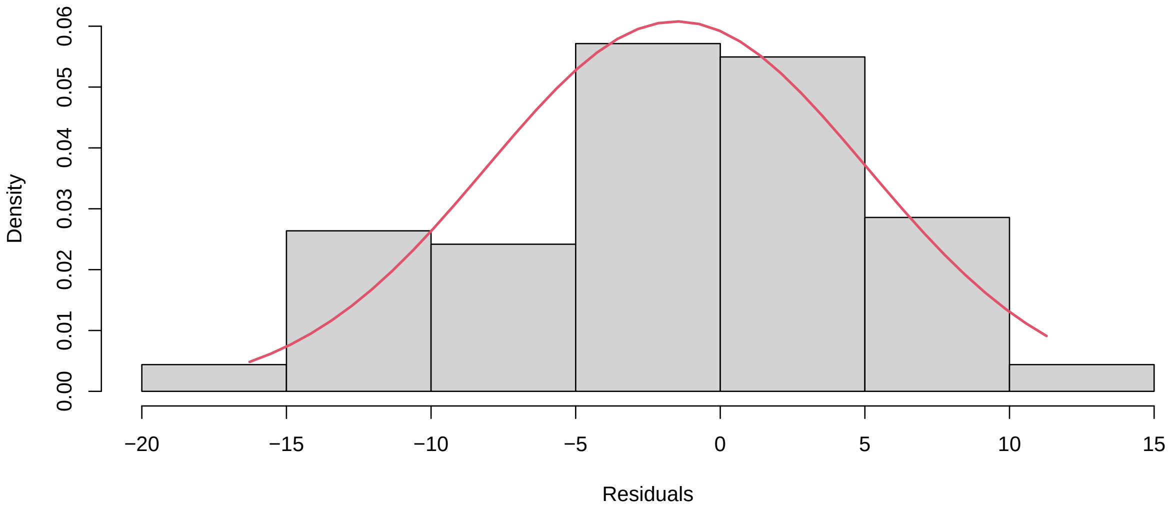

Figure S8.4: Histogram of residuals of the final complex model with co-factors and all POMS subscales. The red solid line shows the density function of normal distribution with parameters calculated from the residuals. The residuals appear to be normally distributed.

Normality of residuals appear to be normally distributed according to histogram above. The Shapiro-Wilk test of normality does not reject the null hypothesis, its p-value is 0.0949709.

S9 Sleep quality trajectory

S9.1 Subjective sleep quality

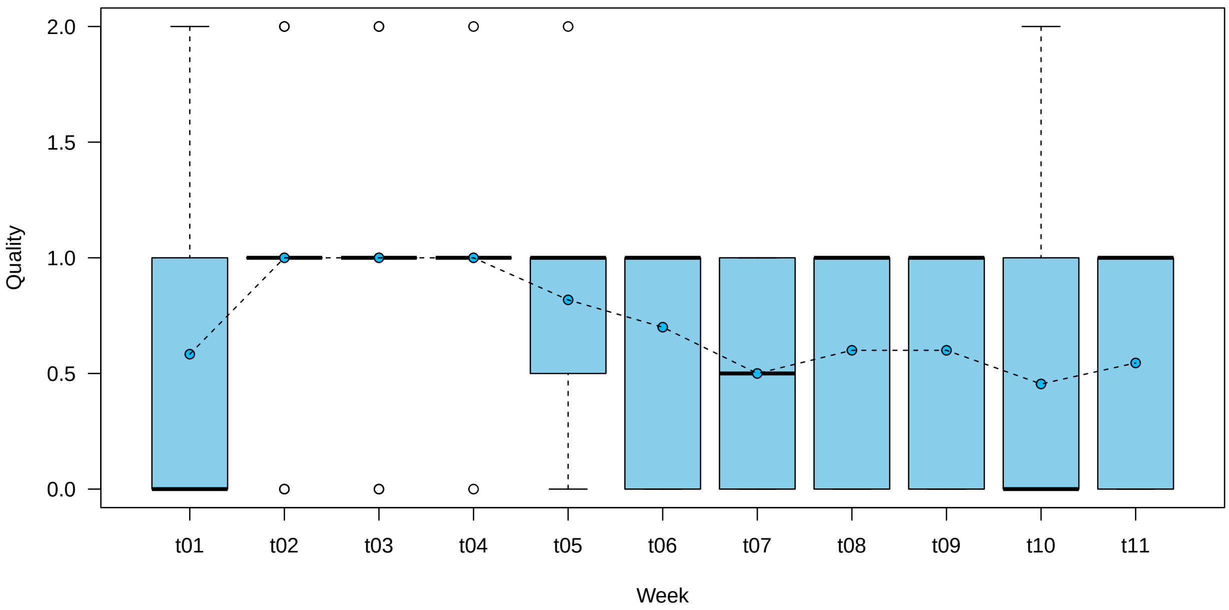

Figure S9.1: Box plots for week-by-week score of PSQI questionnaire (Subjective sleep quality). The mean values are represented by a blue point, median values are represented by a dash.

For modeling the dependence of Subjective sleep quality on time, sex, age, BMI and previous experience with expedition (variable First.timer) we assumed intra-subject correlation of the form AR(1). We compared two models, one assuming homoskedasticity and the other heteroskedasticity, corresponding p-value of the comparison of these two models (using `anova()` function in `R`) is 0.0015063, therefore we continued with the model assuming heteroskedasticity.

Table S9.1: Summary table of the model of dependence of Subjective sleep quality on time, sex, BMI, age and previous experience with expedition (factor First.timer). The values of regression coefficients, their standard errors and corresponding p-values are reported. In the last column adjusted p-values using Benjamini-Hochberg (FDR) correction are shown.

	Value	Std.Error	p-value	p-adjusted
(Intercept)	−0.181817	0.923349	0.844271243	1.000000000
time	−0.017343	0.023283	0.457971899	1.000000000
SexMale	0.618885	0.187827	0.001335441	0.011128677
Age	−0.051895	0.015327	0.000993024	0.011128677
First.timerYes	−0.446982	0.292947	0.130007042	0.812544013
BMI	0.120723	0.032316	0.000302206	0.007555146

S9.2 Sleep latency

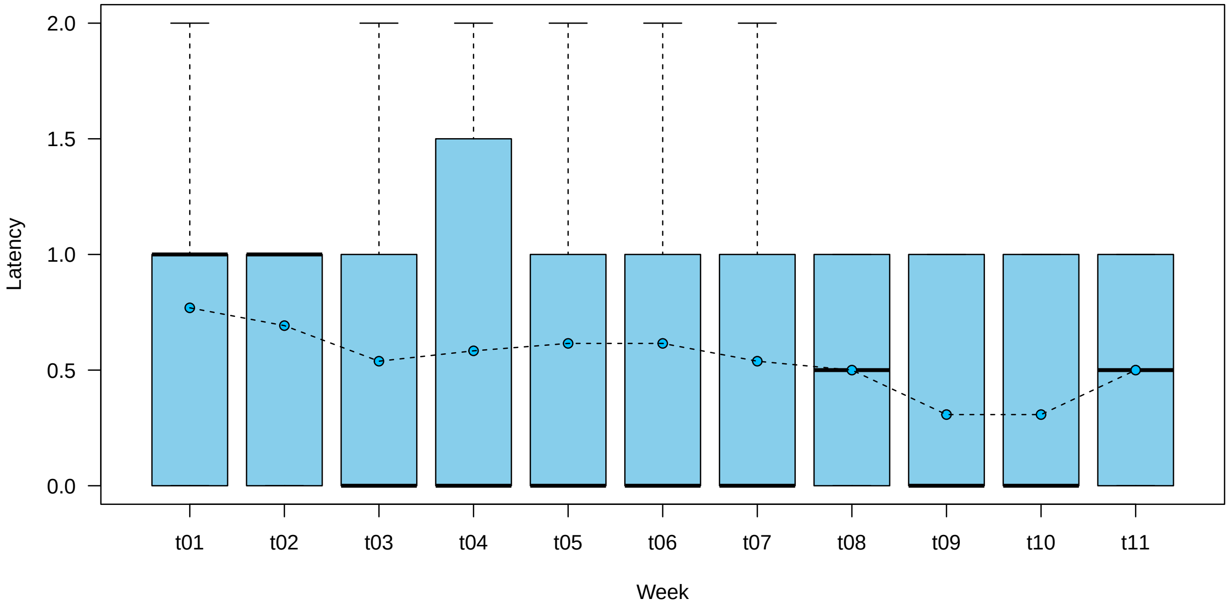

Figure S9.2: Box plots for week-by-week score of PSQI questionnaire (Sleep latency). The mean values are represented by a blue point, median values are represented by a dash.

For modeling the dependence of Sleep latency on time, sex, age, BMI and previous experience with expedition (variable First.timer) we assumed intra-subject correlation of the form AR(1). We compared two models, one assuming homoskedasticity and the other heteroskedasticity, corresponding p-value of the comparison of these two models (using `anova()` function in `R`) is 0.5069346, therefore we continued with the simpler model assuming homoskedasticity.

Table S9.2: Summary table of the model of dependence of Sleep latency on time, sex, BMI, age and previous experience with expedition (factor First.timer). The values of regression coefficients, their standard errors and corresponding p-values are reported. In the last column adjusted p-values using Benjamini-Hochberg (FDR) correction are shown.

	Value	Std.Error	p-value	p-adjusted
(Intercept)	4.008643	1.135043	0.000579059	0.007238233
time	−0.024918	0.020247	0.220750072	1.000000000
SexMale	0.063118	0.215715	0.770312964	1.000000000
Age	−0.065041	0.014723	0.000021377	0.000534422
First.timerYes	−0.973099	0.319314	0.002816490	0.023470754
BMI	−0.019681	0.033430	0.557114062	1.000000000

S10 Mean sleep heart rate and sleep quality

S10.1 All expedition

For modelling the dependence of mean sleep heart rate on both PSQI subscales (Subjective sleep quality and Sleep latency) and other co-factors, we used generalized least squares linear model again. We assumed

different variance of residuals for each subject and also auto-correlation structure of form AR(1) with dependence on time.

```
model.full.1 <- gls(mean_HR ~ Quality + Latency
                    + time + Sex + Age + First.timer + BMI + duration,
                    data = data.full, na.action = na.omit,
                    weights = varIdent(form = ~1|ID),
                    cor = corAR1(form = ~time|ID))
```

Table S10.1: Summary table of the final complex model with co-factors and both PSQI subscales. The values of regression coefficients, their standard errors and corresponding p-values are reported. In the last column adjusted p-values using Benjamini-Hochberg (FDR) correction are shown.

	Value	Std.Error	p-value	p-adjusted
(Intercept)	18.034340	19.961890	0.36838120	1.0000000
Quality	0.157509	0.430136	0.71497081	1.0000000
Latency	0.957713	0.584130	0.10411930	1.0000000
time	−0.233320	0.194880	0.23393139	1.0000000
SexMale	0.218488	4.163443	0.95824862	1.0000000
Age	0.369181	0.361588	0.30962373	1.0000000
First.timerYes	19.321700	7.530425	0.01171837	0.2929592
BMI	0.963471	1.010962	0.34278767	1.0000000
duration	−0.085422	0.276042	0.75759660	1.0000000

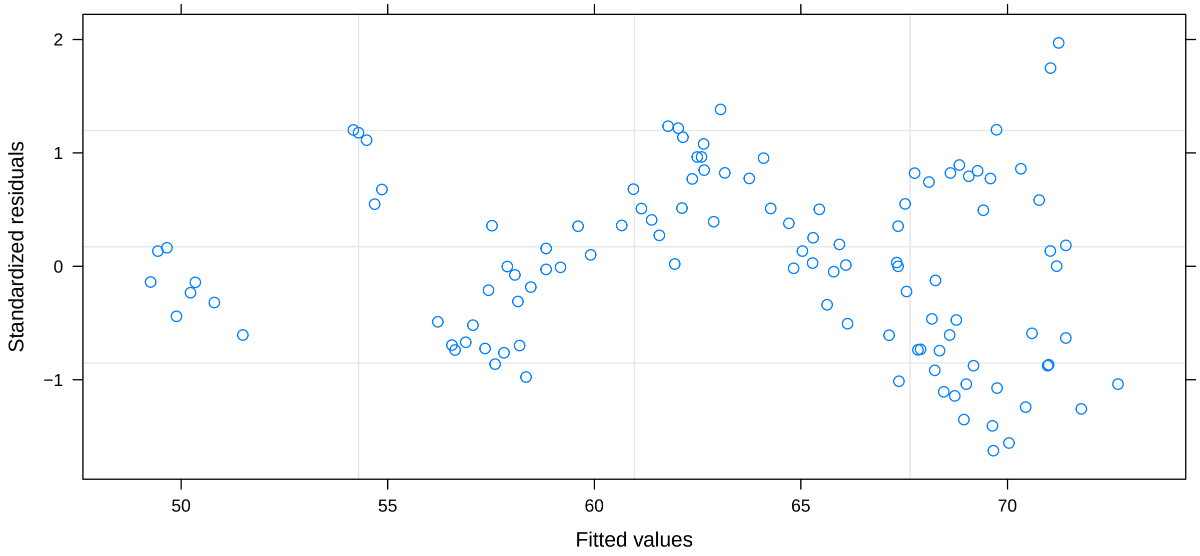

Figure S10.1: Plot of standardized residuals against fitted values. The residuals appear to be randomly distributed around the fitted values.

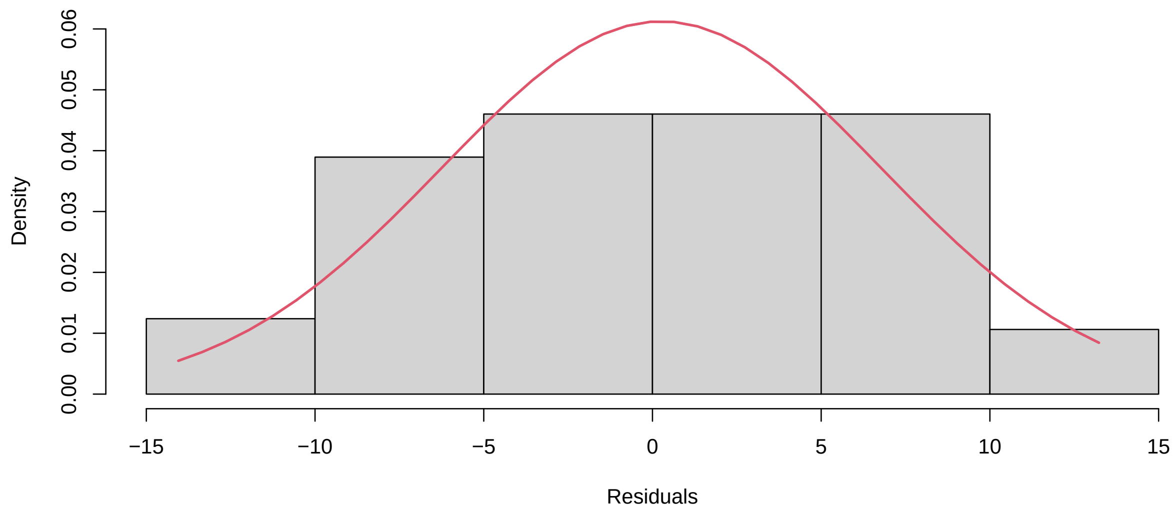

Figure S10.2: Histogram of residuals of the final complex model with co-factors and both PSQI subscales. The red solid line shows the density function of normal distribution with parameters calculated from the residuals. The residuals appear to be normally distributed.

Normality of residuals appear to be normally distributed according to histogram above. The Shapiro-Wilk test of normality does not reject the null hypothesis, its p-value is 0.1393605.

S10.2 Station only

Now we consider only time spent in Antarctica (J. G. Mendel Czech Antarctic Station only). For modelling the dependence of mean sleep heart rate on both PSQI subscales and other co-factors we again used generalized least squares linear model. We assumed different variance of residuals for each subject and also auto-correlation structure of form AR(1) with dependence on time.

```
model.full.2 <- gls(mean_HR ~ Quality + Latency
                    + time + Sex + Age + First.timer + BMI + duration,
                    data = data.reduced.full, na.action = na.omit,
                    weights = varIdent(form = ~1|ID),
                    cor = corAR1(form = ~time|ID))
```

Table S10.2: Summary table of the final complex model with co-factors and both PSQI subscales for J. G. Mendel Czech Antarctic Station only. The values of regression coefficients, their standard errors and corresponding p-values are reported. In the last column adjusted p-values using Benjamini-Hochberg (FDR) correction are shown.

	Value	Std.Error	p-value	p-adjusted
(Intercept)	46.832850	41.239600	0.25987827	1
Quality	0.232616	0.446053	0.60361910	1
Latency	−0.187226	0.851720	0.82663235	1
time	−0.321704	0.182097	0.08152327	1
SexMale	3.543842	8.448468	0.67612479	1
Age	−0.330591	0.567876	0.56228242	1
First.timerYes	10.830080	10.614400	0.31099311	1
BMI	0.874740	1.250962	0.48664664	1
duration	0.281216	0.232440	0.23029718	1

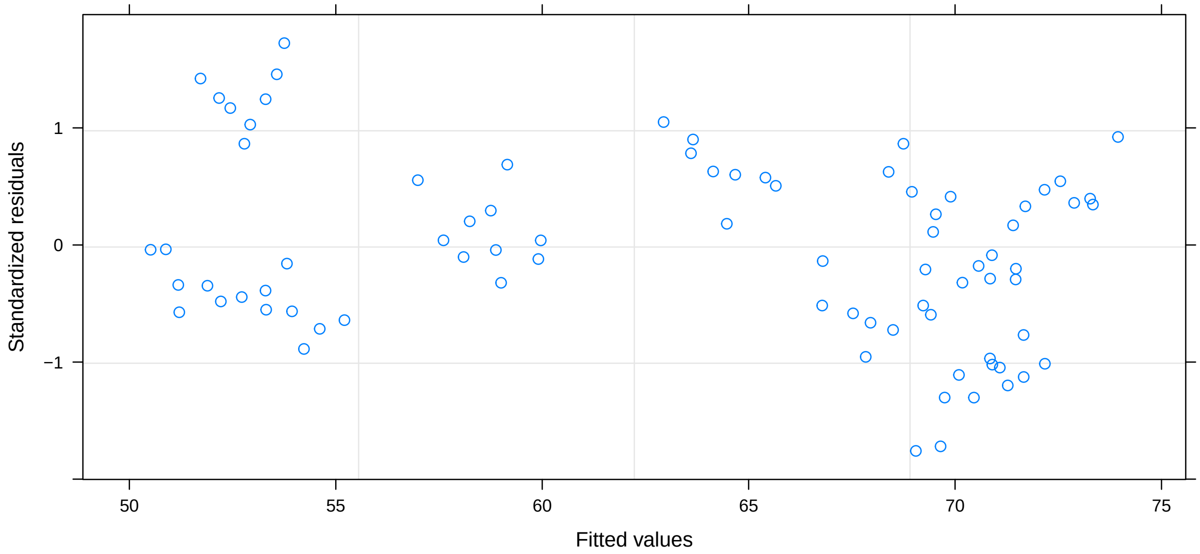

Figure S10.3: Plot of standardized residuals against fitted values. The residuals appear to be randomly distributed around the fitted values.

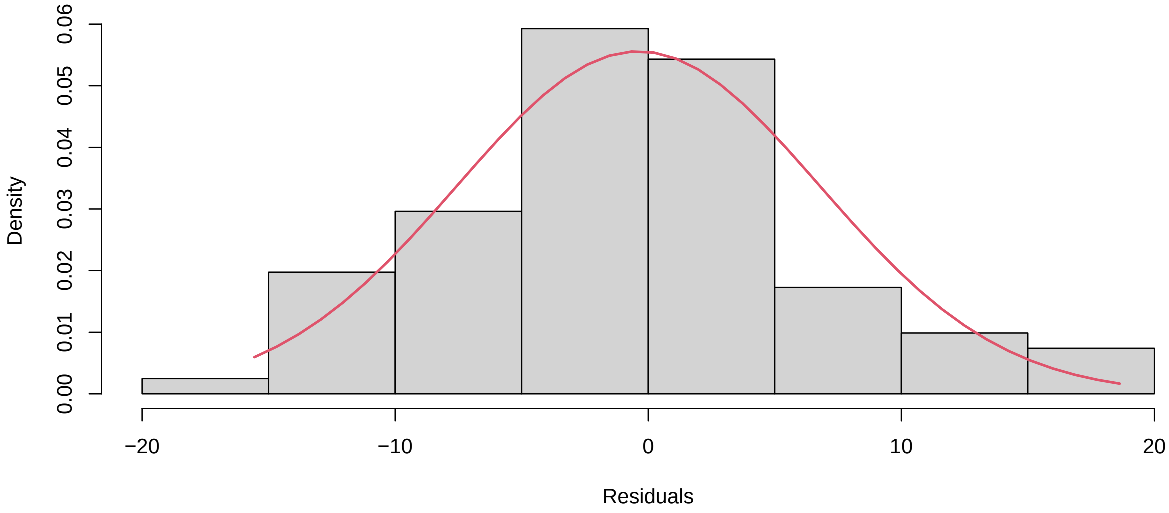

Figure S10.4: Histogram of residuals of the final complex model with co-factors and both PSQI subscales. The red solid line shows the density function of normal distribution with parameters calculated from the residuals. The residuals appear to be normally distributed.

Normality of residuals appear to be normally distributed according to histogram above. The Shapiro-Wilk test of normality does not reject the null hypothesis, its p-value is 0.2699373.

S11 Perceived stress trajectory

S11.1 Perceived helplessness

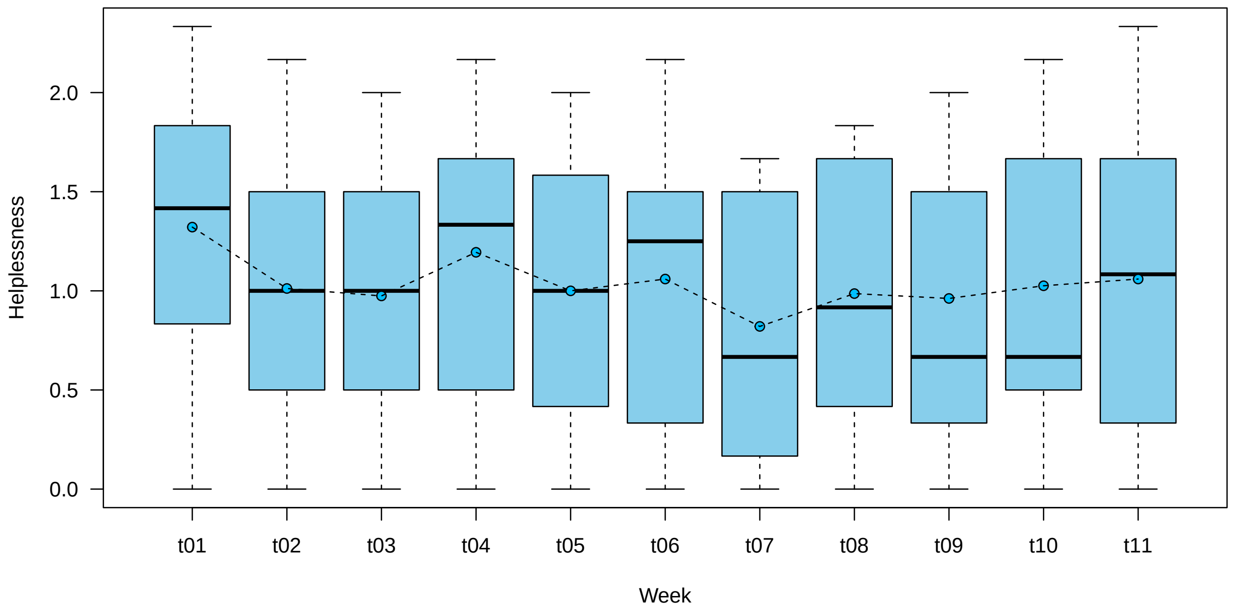

Figure S11.1: Box plots for week-by-week score of PSS-10 questionnaire (Perceived Helplessness). The mean values are represented by a blue point, median values are represented by a dash.

S11.1.1 Simple model with time

For modeling the dependence of Perceived Helplessness on time (we used time as a variable of type `factor`) we assumed intra-subject correlation equal accros all weeks and independence between subjects (Compound symmetry correlation structure). We also assumed homoskedasticity.

```
m.H.week <- gls(Helplessness ~ week,
                data = data.full, na.action = na.omit,
                correlation = corCompSymm(form = ~1|ID))
```

We used `anova()` function to decide whether there is a statistically significant difference between weeks. The p-value is 0.2187161, therefore simple model with time as a factor did not reveal any statistically significant differences between weeks.

S11.1.2 Complex model with co-factors

For modeling the dependence of Perceived Helplessness on time, sex, age, BMI and previous experience with expedition (variable First.timer) we assumed intra-subject correlation of the form AR(1). We compared two models, one assuming homoskedasticity and the other heteroskedasticity, corresponding p-value of the comparison of these two models is 0.2538635, therefore we continued with the simpler model assuming homoskedasticity.

Table S11.1: Summary table of the model of dependence of Perceived Helplessness on time, sex, BMI, age and previous experience with expedition (factor First.timer). The values of regression coefficients, their standard errors and corresponding p-values are reported. In the last column adjusted p-values using Benjamini-Hochberg (FDR) correction are shown.

	Value	Std.Error	p-value	p-adjusted
(Intercept)	−3.139153	1.679488	0.063910411	0.45076127
time	−0.027347	0.023052	0.237704329	1.00000000
SexMale	−0.922834	0.322982	0.004995168	0.06243961
Age	0.023428	0.021834	0.285294061	1.00000000
First.timerYes	0.851930	0.469781	0.072121803	0.45076127
BMI	0.158974	0.049584	0.001701851	0.04254628

S11.2 Lack of self efficacy

S11.2.1 Simple model with time

For modeling the dependence of Lack of self efficacy on time (we used time as a variable of type `factor`) we assumed intra-subject correlation equal accros all weeks and independence between subjects (Compound symmetry correlation structure). We also assumed homoskedasticity.

```
m.E.week <- gls(Efficacy ~ week,
                data = data.full, na.action = na.omit,
                correlation = corCompSymm(form = ~1|ID))
```

We used `anova()` function to decide whether there is a statistically significant difference between weeks. The p-value is 0.644181, therefore simple model with time as a factor did not reveal any statistically significant differences between weeks.

S11.2.2 Complex model with co-factors

For modeling the dependence of Lack of self efficacy on time, sex, age, BMI and previous experience with expedition (variable First.timer) we assumed intra-subject correlation of the form AR(1). We compared two models, one assuming homoskedasticity and the other heteroskedasticity, corresponding p-value of the comparison of these two models is 0.4558137, therefore we continued with the simpler model assuming homoskedasticity.

Table S11.2: Summary table of the model of dependence of Lack of self efficacy on time, sex, BMI, age and previous experience with expedition (factor First.timer). The values of regression coefficients, their standard errors and corresponding p-values are reported. In the last column adjusted p-values using Benjamini-Hochberg (FDR) correction are shown.

	Value	Std.Error	p-value	p-adjusted
(Intercept)	−0.573004	1.498239	0.70277056	1.0000000
time	0.004979	0.025135	0.84327704	1.0000000
SexMale	−0.684776	0.287949	0.01890429	0.4580297
Age	−0.005175	0.019557	0.79173005	1.0000000
First.timerYes	0.216493	0.422585	0.60933357	1.0000000
BMI	0.093363	0.044201	0.03664238	0.4580297

S11.3 Mean total score

S11.3.1 Simple model with time

For modeling the dependence of Mean total score on time (we used time as a variable of type `factor`) we assumed intra-subject correlation equal accros all weeks and independence between subjects (Compound symmetry correlation structure). We also assumed homoskedasticity.

```
m.PS.week <- gls(PSS.score ~ week,
                 data = data.full, na.action = na.omit,
                 correlation = corCompSymm(form = ~1|ID))
```

We used `anova()` function to decide whether there is a statistically significant difference between weeks. The p-value is 0.404476, therefore simple model with time as a factor did not reveal any statistically significant differences between weeks.

S11.3.2 Complex model with co-factors

For modeling the dependence of Mean total score on time, sex, age, BMI and previous experience with expedition (variable First.timer) we assumed intra-subject correlation of the form AR(1). We compared two models, one assuming homoskedasticity and the other heteroskedasticity, corresponding p-value of the comparison of these two models is 0.6827547, therefore we continued with the simpler model assuming homoskedasticity.

Table S11.3: Summary table of the model of dependence of Mean total score on time, sex, BMI, age and previous experience with expedition (factor First.timer). The values of regression coefficients, their standard errors and corresponding p-values are reported. In the last column adjusted p-values using Benjamini-Hochberg (FDR) correction are shown.

	Value	Std.Error	p-value	p-adjusted
(Intercept)	−22.214970	15.261430	0.147982522	0.92489076
time	−0.130119	0.196929	0.509984670	1.00000000
SexMale	−8.419447	2.935518	0.004842359	0.06052949
Age	0.127932	0.198253	0.519908865	1.00000000
First.timerYes	6.305366	4.260542	0.141384046	0.92489076
BMI	1.360341	0.450816	0.003084496	0.06052949

S12 Mean sleep heart rate and perceived stress

For modelling the dependence of mean sleep heart rate on both PSS-10 subscales (Perceived Helplessness and Lack of self efficacy) and other co-factors we used generalized least squares model. We assumed different variance of residuals for each subject and also auto-correlation structure of form AR(2) or AR(1) respectively with dependence on time. Now we model the dependence of mean sleep heart rate on time assuming linear trend for all expedition and quadratic trend for J. G. Mendel Czech Antarctic Station only, because the quadratic time covariate has statistically significant influence on the mean sleep heart rate in this case. Also the diagnostics appear to be better and quadratic model gives better results.

S12.1 All expedition

```
model.full.1 <- gls(mean_HR ~ Helplessness + Efficacy
                    + time + Sex + Age + First.timer + BMI + duration,
                    data = data.full, na.action = na.omit,
                    weights = varIdent(form = ~1|ID),
                    cor = corARMA(p = 2, form = ~time|ID))
```

Table S12.1: Summary table of the final complex model with co-factors and both PSS-10 subscales. The values of regression coefficients, their standard errors and corresponding p-values are reported. In the last column adjusted p-values using Benjamini-Hochberg (FDR) correction are shown.

	Value	Std.Error	p-value	p-adjusted
(Intercept)	35.411200	30.509480	0.24804538	1.0000000
Helplessness	−0.684523	0.504954	0.17772606	0.8886303
Efficacy	0.822414	0.409420	0.04677377	0.5846721
time	−0.253517	0.152226	0.09840019	0.6150012
SexMale	0.981449	6.479404	0.87985349	1.0000000
Age	0.114289	0.422538	0.78724576	1.0000000
First.timerYes	16.919250	8.021883	0.03697728	0.5846721
BMI	0.794417	0.874545	0.36546936	1.0000000
duration	−0.490215	0.271082	0.07301305	0.6084421

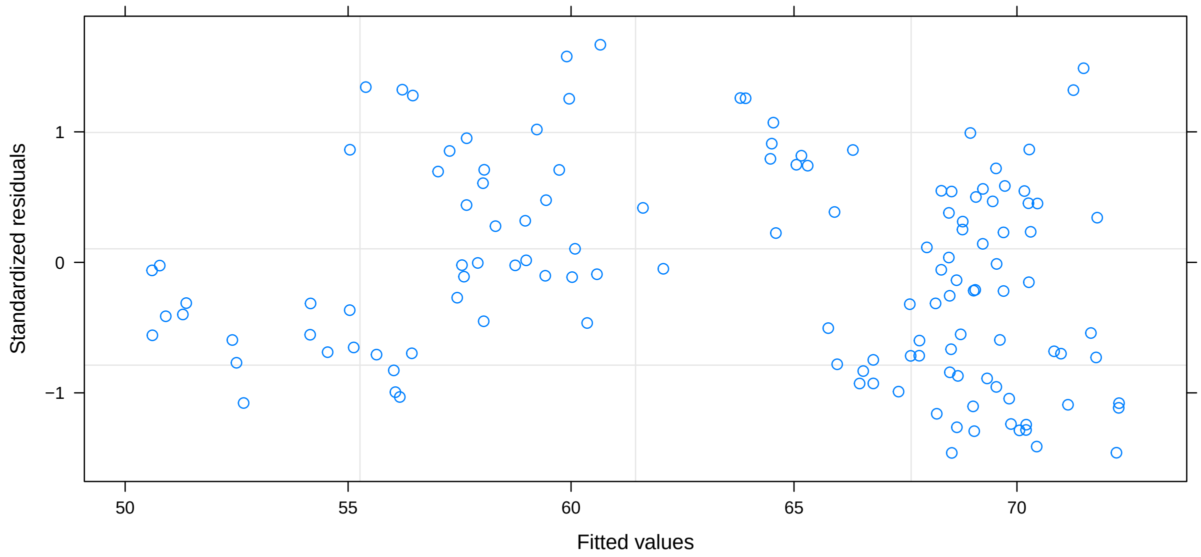

Figure S12.1: Plot of standardized residuals against fitted values. The residuals appear to be randomly distributed around the fitted values.

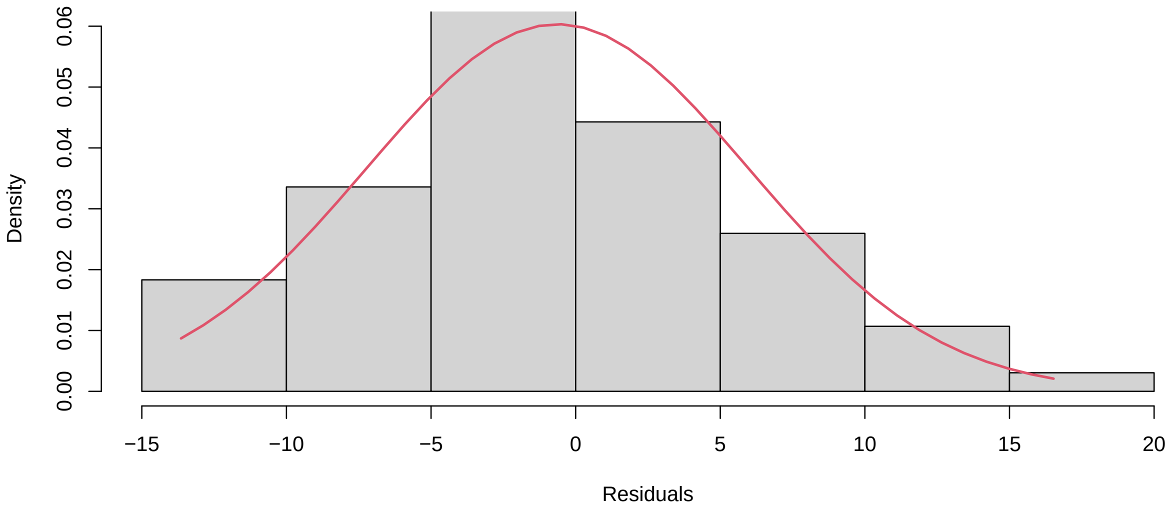

Figure S12.2: Histogram of residuals of the final complex model with co-factors and both PSS-10 subscales. The red solid line shows the density function of normal distribution with parameters calculated from the residuals. The residuals appear to be normally distributed.

Normality of residuals appear to be normally distributed according to histogram above. The Shapiro-Wilk test of normality does not reject the null hypothesis, its p-value is 0.1649736.

S12.2 Station only

Now we consider only time spent in Antarctica (J. G. Mendel Czech Antarctic Station only). For modelling the dependence of mean sleep heart rate on both PSS-10 subscales and other co-factors we again used generalized least squares linear model. We assumed different variance of residuals for each subject and also auto-correlation structure of form AR(1) with dependence on time.

```
model.full.2 <- gls(mean_HR ~ Helplessness + Efficacy
                    + time + I(time^2) + Sex + Age + First.timer + BMI + duration,
                    data = data.reduced.full, na.action = na.omit,
                    weights = varIdent(form = ~1|ID),
                    cor = corAR1(form = ~time|ID))
```

Now we assumed quadratic dependence of mean sleep heart rate on time, because the p-value and also adjusted p-value using FDR correction of quadratic time covariate significance are below the significance level 0.05. We can use `anova()` function in `R` to compare linear and quadratic models, the corresponding p-value is 0.0156466

Table S12.2: Summary table of the final complex model with co-factors and both PSS-10 subscales for J. G. Mendel Czech Antarctic Station only. The values of regression coefficients, their standard errors and corresponding p-values are reported. In the last column adjusted p-values using Benjamini-Hochberg (FDR) correction are shown.

	Value	Std.Error	p-value	p-adjusted
(Intercept)	32.251960	24.962250	0.199932846	1.000000000
Helplessness	0.080662	0.547096	0.883144622	1.000000000
Efficacy	0.102687	0.490711	0.834757304	1.000000000
time	−3.016669	0.795133	0.000280716	0.005610315
I(time^2)	0.211838	0.057949	0.000448825	0.005610315
SexMale	0.490024	5.286617	0.926372115	1.000000000
Age	0.280274	0.323772	0.389176197	1.000000000
First.timerYes	18.848640	6.438576	0.004408852	0.036740433
BMI	0.843684	0.734479	0.253986177	1.000000000
duration	−0.076994	0.234068	0.743031900	1.000000000

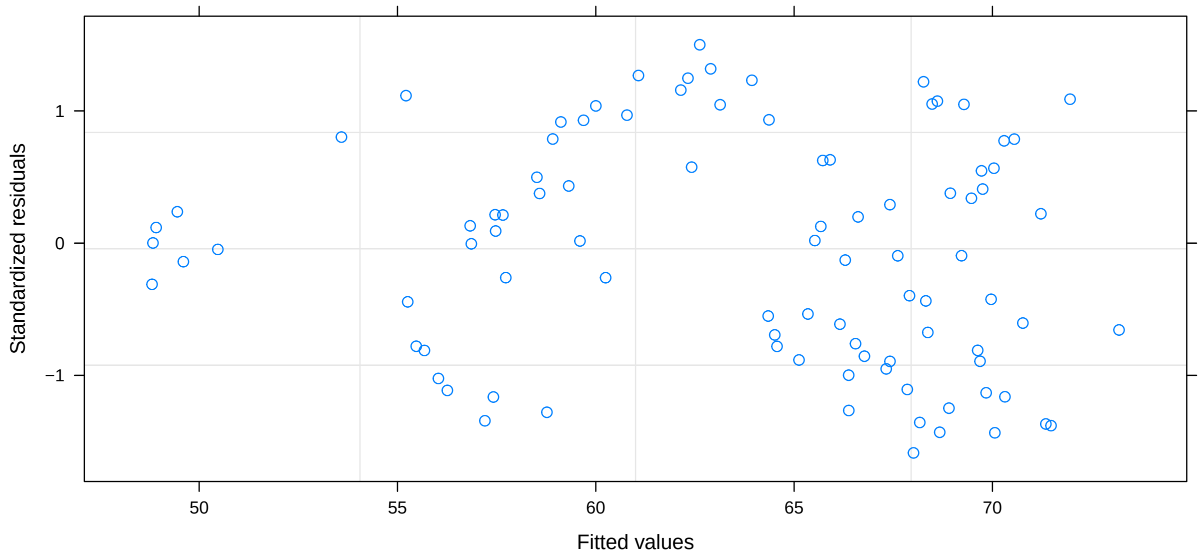

Figure S12.3: Plot of standardized residuals against fitted values. The residuals appear to be randomly distributed around the fitted values.

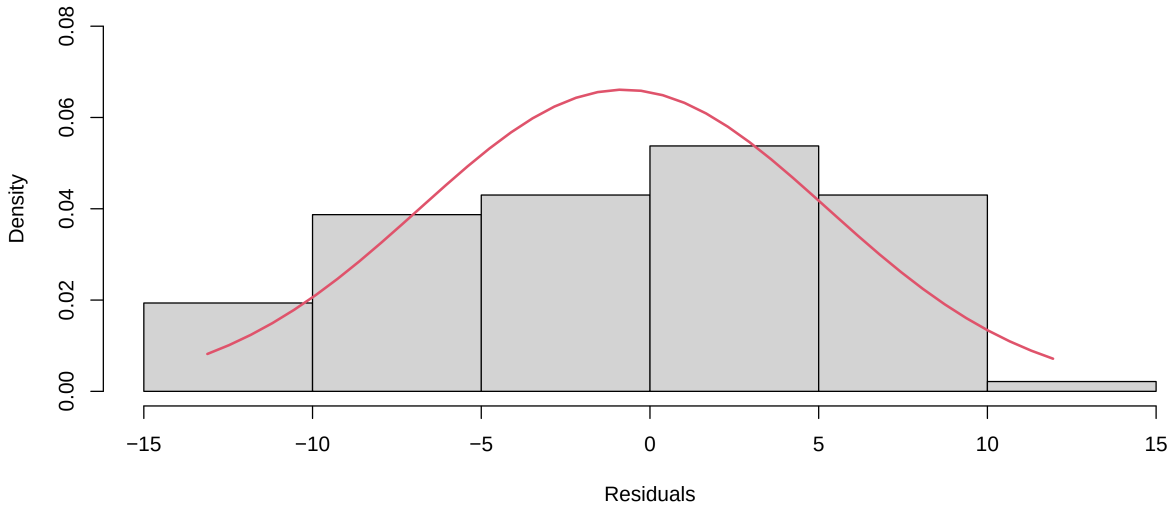

Figure S12.4: Histogram of residuals of the final complex model with co-factors and both PSS-10 subscales. The red solid line shows the density function of normal distribution with parameters calculated from the residuals. The residuals appear to be normally distributed.

Normality of residuals appear to be normally distributed according to histogram above. The Shapiro-Wilk test of normality does not reject the null hypothesis, its p-value is 0.0937205.

S13 P-value adjustment

Throughout the whole analysis we set the significance level $\alpha = 0.05$, which controls the probability of Type I error. Due to the large number of models reported in the main article and in the sections above, we had to control the rate of Type I errors in null hypothesis testing by adjusting the p-values. We selected Benjamini-Hochberg procedure (false discovery rate method – FDR) because it is “less conservative and more appropriate approach for identifying the important few from the trivial many effects tested” (for more details see wikipedia.org[3] or article[4] written by Benjamini and Hochberg).

We also chose to adjust the p-values not to the number of all tests we had done during whole analysis, instead we adjusted the p-values to the number of models reported in the main article (this number is 25 models). For computing adjusted p-values using Benjamini-Hochberg FDR procedure we used `p.adjust()` function from `stats` library in `R`. We chose the method `method = 'fdr'` and set the argument `n = 25`. The code than looked as follows:

```
p.adj <- summary(model)$tTable[, 'p-value'] |>
  p.adjust(method = 'fdr', n = 25)
```

In this Supplementary document and also in the main article, we report for all models corresponding p-values of null hypothesis about significance of effects of individual covariates before adjustment alongside with the adjusted p-values using Benjamini-Hochberg procedure adjusting for the number of 25 models.

[3]Wikipedia contributors. (2023, June 19). False discovery rate. In Wikipedia, The Free Encyclopedia. Retrieved 12:02, August 2, 2023, from https://en.wikipedia.org/w/index.php?title=False_discovery_rate&oldid=1160968238

[4]Benjamini, Y., & Hochberg, Y. (1995). Controlling the False Discovery Rate: A Practical and Powerful Approach to Multiple Testing. Journal of the Royal Statistical Society. Series B (Methodological), 57(1), 289–300. http://www.jstor.org/stable/2346101
